# Saccade learning with concurrent cortical and subcortical basal ganglia loops

**DOI:** 10.3389/fncom.2014.00048

**Published:** 2014-04-23

**Authors:** Steve N'Guyen, Charles Thurat, Benoît Girard

**Affiliations:** ^1^Sorbonne Universités, UPMC Univ Paris 06, UMR 7222, ISIRParis, France; ^2^CNRS, UMR 7222, ISIRParis, France; ^3^LPPA, Collège de France, CNRS UMR 7152Paris, France

**Keywords:** basal ganglia, superior colliculus, saccades, decision making, reinforcement learning

## Abstract

The Basal Ganglia (BG) is a central structure involved in multiple cortical and subcortical loops. Some of these loops are believed to be responsible for saccade target selection. We study here how the very specific structural relationships of these saccadic loops can affect the ability of learning spatial and feature-based tasks. We propose a model of saccade generation with reinforcement learning capabilities based on our previous BG and superior colliculus models. It is structured around the interactions of two parallel cortico-basal loops and one tecto-basal loop. The two cortical loops separately deal with spatial and non-spatial information to select targets in a concurrent way. The subcortical loop is used to make the final target selection leading to the production of the saccade. These different loops may work in concert or disturb each other regarding reward maximization. Interactions between these loops and their learning capabilities are tested on different saccade tasks. The results show the ability of this model to correctly learn basic target selection based on different criteria (spatial or not). Moreover the model reproduces and explains training dependent express saccades toward targets based on a spatial criterion. Finally, the model predicts that in absence of prefrontal control, the spatial loop should dominate.

## 1. Introduction

The basal ganglia (BG) are a set of interconnected subcortical nuclei (Redgrave, 2007), which are thought to be central in the performance of action selection (Mink, 1996; Redgrave et al., 1999).

**Figure 1:**
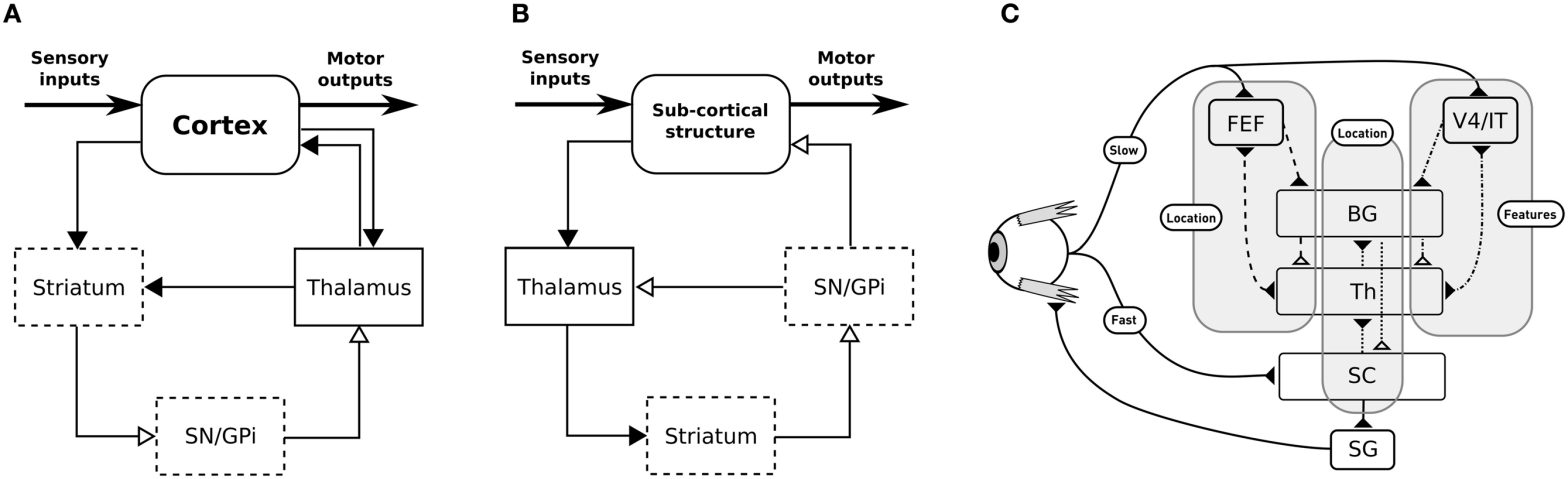
**(A)** General organization for cortical loops. **(B)** General organization for subcortical loops. Filled arrow heads are exitatory connexions, empty arrow heads are inhibitory connexions. Dashed block are inhibitory structures. Note that the concerned thalamus nuclei are differents between **(A)** (ventral anterior, ventrolateral, medial dorsal) and **(B)** (pulvinar, lateral posterior, rostral and caudal intralaminar). **(A,B)** Adapted from McHaffie et al. (2005). **(C)** Schematic representation of the relationships between the three modeled loops, note the type of information processed (either location or features of targets) and the delays (slow or fast).

The BG are traditionally described as being composed of various parallel subcircuits with identical internal wiring, implied in different functions (from motor to cognitive ones), and belonging to a set of parallel cortico-baso-thalamo-cortical loops (Alexander et al., 1986), as schematized in Figure 1A. However, the BG also participate in purely subcortical loops (Groenewegen and Berendse, 1994; McHaffie et al., 2005, 2006; May, 2006), which are wired a bit differently as the input to the BG is relayed through the thalamus and the BG output projects directly to the considered subcortical structures (Figure 1B), and which rely on different thalamic nuclei (pulvinar, lateral posterior, rostral, and caudal intralaminar). They do, in particular, participate in loops with the superior colliculus (SC), well-known for its laminar structure, its mapping of the visual field and its involvement in gaze orientation movements, including saccadic eye movements (Moschovakis et al., 1996; Lynch and Tian, 2006).

We propose here a computational model of the interactions of subcortical and cortical BG loops in primates, processing either target position (spatial) information or target feature information, in the well investigated framework of saccadic eye movements (Hikosaka et al., 2000). Indeed, cortico-basal loops dealing with the location of potential targets in the visual field, on the one hand, or with the detection of features of potential targets, on the other hand, have long been identified. The SC (and thus the tecto-basal loop) is a bottleneck receiving all this information for the final decision, however it also receives target location information earlier than the cortically processed information, through direct projections from the retina.

We thus study the effects imposed by this hierarchical structure—where the highest level modules have longer latencies, while the lowest level module has a lower latency shortcut, but specific to location information, Figure 1C—on performance and saccadic reaction time in space-based and/or feature-based selection tasks, in order to identify predictions specific to this organization. These predictions stand for dorsolateral prefrontal cortex (dlPFC) deprived animals as it is not included in our model and as we can expect the inhibitory control from the dlPFC on the SC to allow additional control on unwanted short-latency saccades (Koval et al., 2011).

We show that the fact that a purely spatial selection and learning system operate at the last level predicts that:

- in spatial tasks only should the saccadic reaction times decrease with learning, allowing the generation of express saccades and causing short latency activations in the FEF,
- performance in feature-based tasks should be lower than in spatial tasks, because of the perturbations caused by the subcortical spatial loop,
- in conjunction tasks, where spatial and feature-based information determine the good choice, errors are unavoidable when no choice should be made.

## 2. Materials and methods

### 2.1. Global architecture

**Figure 2:**
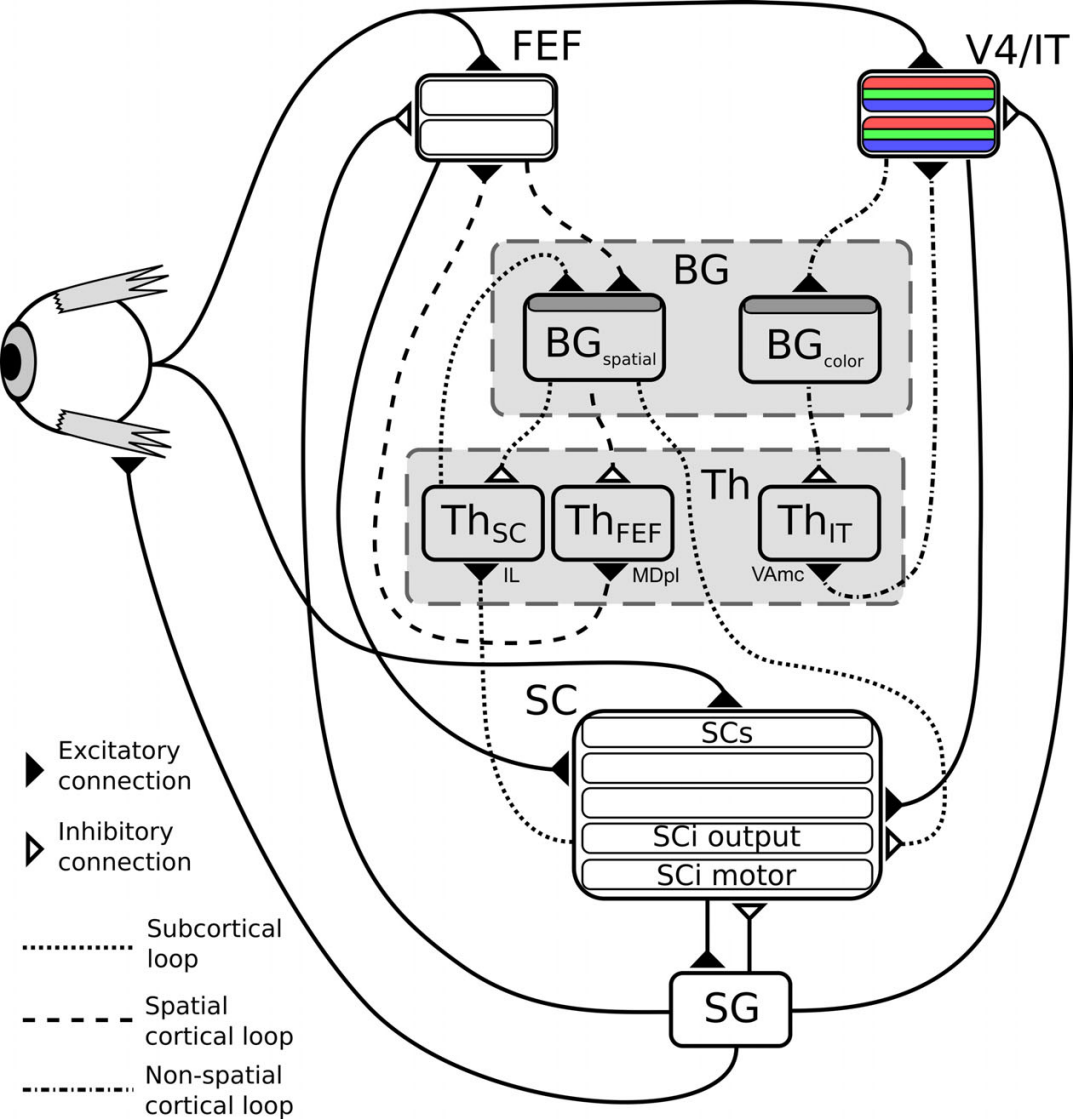
**Structure of the model**. BG, Basal ganglia; FEF, frontal eye fields; SG, saccade generators; SC, superior colliculus; Th, thalamus; V4|IT, Feature perception area including IT (TE region) interacting with V4 visual cortex area. Dark gray shaded layers on BG modules are input layers with reinforcement learning capabilities.

The subcortical loop (Figure 2, dotted circuit) has access to visual inputs directly conveyed from the retina to the superficial layers of the SC, with a low latency. These retinal projections provide relatively rich visual information (Girman and Lund, 2007), but no color information. As the SC layers are organized as piled retinotopic maps of the visual field, and given the spatial receptive fields of the BG output neurons projecting to the SC (Hikosaka and Wurtz, 1983), it can be assumed that the competition among targets is here based on spatial position. This loop is a good candidate neural substrate to explain the accumulating evidence [see for example McPeek and Keller (2002); McPeek et al. (2003); McPeek and Keller (2004), among many others since 2000] that the SC performs target selection on its own, rather than solely executing cortical decisions.

Two cortical loops, projecting to the SC as a common output, are considered. A first one (Figure 2, dashed circuit), comprising the frontal eye fields (FEF), also operates on the spatial domain, but contributes to saccade generation with longer latencies than the SC. This loop is known to be a common pathway for “cognitive” saccades, where working memory or sequence generation are involved, however these are not included in the proposed model (indeed no SEF and pre-SEF have been included). We hypothesize that the BG subcircuit involved in this loop is shared with the subcortical one (i.e., there is only one BG subcircuit dedicated to spatial selection of targets). This choice of converging input has been made based on known anatomy as it seems that FEF projects to the “Oculomotor Striatum” (central/longitudinal Caudate) (Stanton et al., 1988).

The second one (Figure 2, dash-dot circuit) comprises V4 and IT and deals with the selection of targets exhibiting specific features (only color will be used here for simplicity). V4 is known to be selective to shape and color (Ogawa and Komatsu, 2004) and visuotopically organized (Gattass et al., 1988). Moreover, this region exhibits strong recurrent connections with IT (in particular the TE area) (Ungerleider et al., 2008). The TE region of IT has been shown to be selective to features (and colors) and not visuotopically organized (activity doesn't depend on object position) (Tompa and Sáry, 2010). More importantly, this TE area forms a loop with the BG (Middleton and Strick, 1996), thus it seems somewhat reasonable to hypothesize that colors and features could be selected through a cortical IT-BG-Th loop in a non-spatial fashion and then projected back to V4. In particular, the TE region, projects to the “Visual Striatum” (tail of Caudate and caudal/ventral portion of Putamen) (Middleton and Strick, 1996), supporting the separation between the spatial and the feature loop. The SC is known to receive numerous projections from cortical areas amongst which V4 (Fries, 1984; Lock et al., 2003). This mechanism is compatible with feature/color sensitivity with a longer latency than luminance signal observed in intermediate layers of SC (SCi) (White et al., 2009; White and Munoz, 2011).

So to summarize, in this model two parallel mechanisms compete for target selection (Figure 1C). The first one is “location” based and comprises two cooperating loops, both cortical and subcortical. The second one is “feature” based and comprises one cortical loop. The detail of the equations are given below.

### 2.2. Model description

The proposed model is intended to learn to generate saccades toward targets selected based on their color and location in the visual field (cf. Figure 2), depending on the reward contingencies experienced during interaction with the environment.

As said before, it is composed of three main loops going through the BG, which interact in both competitive and cooperative ways. The subcortical one corresponds to the SC-Th-BG circuit (dotted connexions on Figure 2), it gets its inputs from the direct projections from the retina to the superficial layers of the superior colliculus along with activity of deep layers, and it selects among targets competing on a purely spatial dimension. This loop passes through the Intralaminar nucleus (IL) thalamic relay (McHaffie et al., 2005).

The cortical ones also comprise a circuit dedicated to spatial competition (FEF-BG-Th, dashed connexions on Figure 2), which shares its BG circuit with the subcortical loop but with a different thalamic relay (the paralamellar portion of the mediodorsal thalamic nuclei, MDpl) (Alexander et al., 1986; Tian and Lynch, 1997) and another dedicated to features (namely color) selection (IT-BG-Th, dot-dashed connexions on Figure 2) via VAmc (Middleton and Strick, 1996).

Retinal information is transmitted to SC, FEF, and V4|IT with different latencies according to the literature, SCs input latency is fixed to 41 ms (type I neurons) (Rizzolatti and Buchtel, 1980). FEF to 91 ms and IT to 122 ms (average over all TE sub-regions) (Lamme and Roelfsema, 2000).

The FEF module contains an input and an output retinotopic map sensible to luminance. The V4|IT module contains one input and one output retinotopic maps for each color. SC module also contains several retinotopic maps, dealing with direct retinal input (SCs), FEF input, V4|IT input, summed activity of SCs, FEF and V4|IT (SCi output) and motor activity (SCi motor). For each of these structures a selection loop through BG occurs.

We use rate-coding models of neurons [based on locally projected dynamical systems, lPDS, (Girard et al., 2008)], which are defined as follows:

(1) $$\dot{x}=\Pi_{\left[0,max\right]}\left(x(t),\frac{I(t)-x(t)}{\tau }\right)$$

where *I*(*t*) represents the external inputs, τ the time constant, and Π_[0, *max*]_ a projection operator ensuring that the neuron activity *x*(*t*) will remain within [0, *max*].

The projection operator Π_[0, *max*]_ is simply an operator acting on ẋ ensuring that the variable *x* remains within a specified range of values. In our case (Euler integration with 1 ms timestep) we end up with a discrete update operating as follows :

(2) $$x(t+dt)=min\left[1,max\left[0,x(t)+\frac{dt}{\tau }\times (I(t)-x(t))\right]\right]$$

This method is very similar to the classical way of converting the computed activity *x* into a non-negative one *y* = *max*(0.0, *x*) but here the non-linear “transfer function” is applied inside the differential equation at the cost of making it a non-longer a classical ordinary differential equation but with some over benefits such as “contraction” i.e., stability.

The BG model we use here (Girard et al., 2008) was formulated in this framework, so as to formally ensure its dynamical stability. For the sake of consistency, we thus use it for the rest of the model presented here. Only the external input part [*I*(*t*)] and the time constant (τ) of this equation have to be specified to define such a neuron model. Thus, to simplify the writing, only *I*(*t*) will be given in the next section providing a detailed description of the model, while the time constants and other model parameters are provided in supplemental data section.

The BG exert an inhibitory influence on their target circuits, which prevents them from generating actions. Even without any inputs, the BG converge to a given level of inhibition, *GPi|SNr*_rest_, sufficient to enforce this control. As previously proposed in Arai et al. (1994, 1999); Das et al. (1996), we modeled the effect of the basal ganglia inhibition as modulating the excitatory inputs of the targeted systems. To ensure that, at rest, no action can be generated, this inhibitory gain modulation is normalized with regards to the *GPi|SNr*_rest_ constant. Thus, the contributions of the BG outputs to the circuits they target will take the general following form in the equations of the next section:

(3) $$W_{E}\times I_{E}\times \left(1-\frac{GPi|SNr}{GPi|SNr_{rest}}\right)$$

Where *I*_*E*_ is the excitatory input controlled by the BG inhibition, *GPi|SNr* is the output of the BG neurons projecting to the considered circuit.

The feedback from the SC, which signals the end of the execution of a saccade, is also modeled as modulating.

Most of the components of the model are 70 × 70 2D maps of lPDS neurons for each hemifield, respecting the complex-logarithmic geometry of the macaque SC, as modeled by Ottes et al. (1986). Unless specified, neurons of one map project to those of another map in a one-to-one manner. Visual inputs are simulated as gaussian activities spreading over a hundred of neurons.

#### 2.2.1. Cortical and subcortical loops

**Figure 3:**
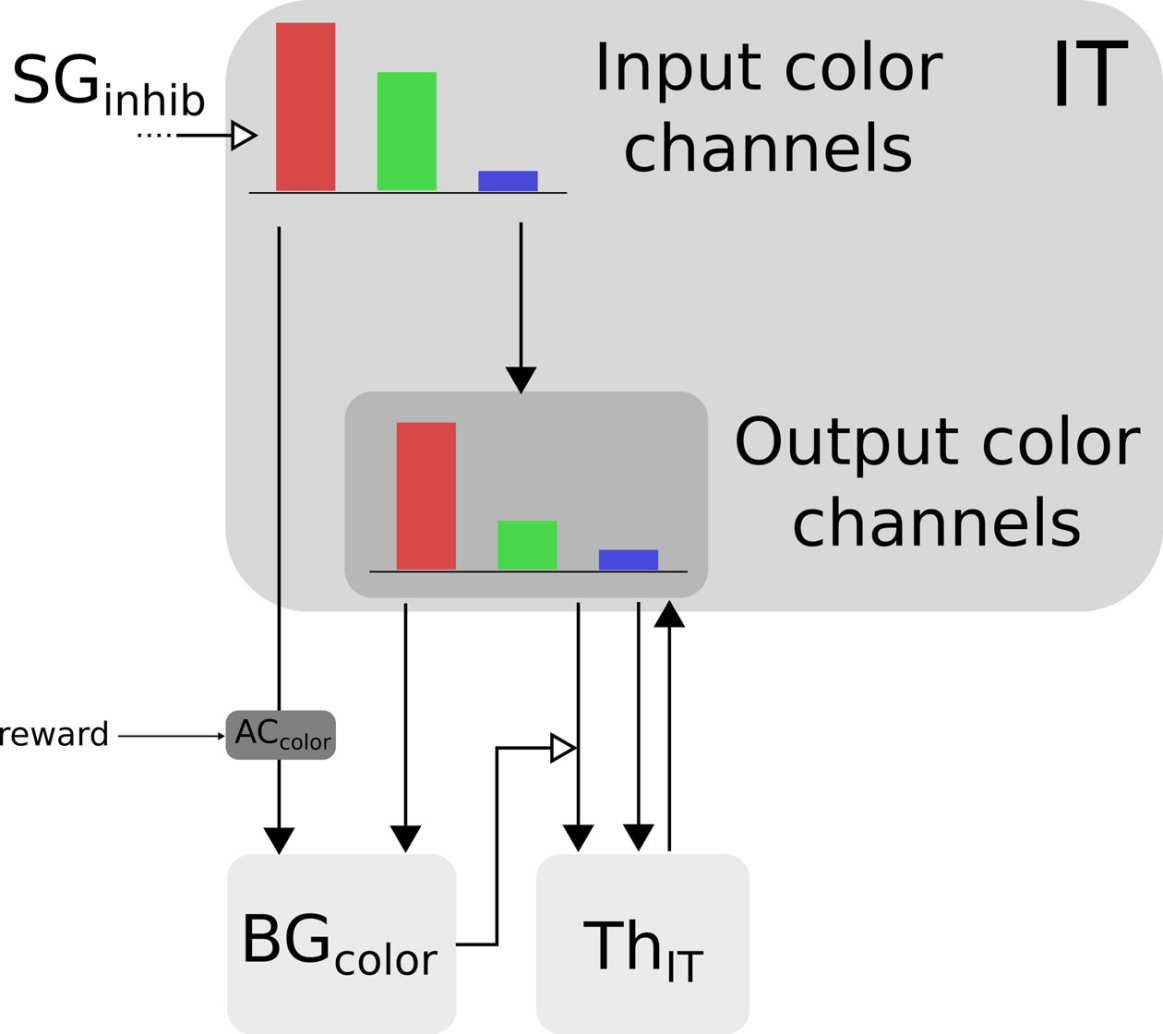
**Selection loop for color channels**. Black arrow heads are exitatory connexions, empty arrow heads are inhibitory connexions.

Color information is processed by the cortical V4|IT-BG-Th loop. As stated previously, the V4 structure contains several retinotopic maps each encoding for a specific color (3 are used here, red, green, and blue). In order to deal with non-spatial color channels, activity in each map is summed, providing a reduced number of independent channel. These channels are amplified in an closed loop manner by the interaction of IT with BG and Th (Figure 3).

**Figure 4:**
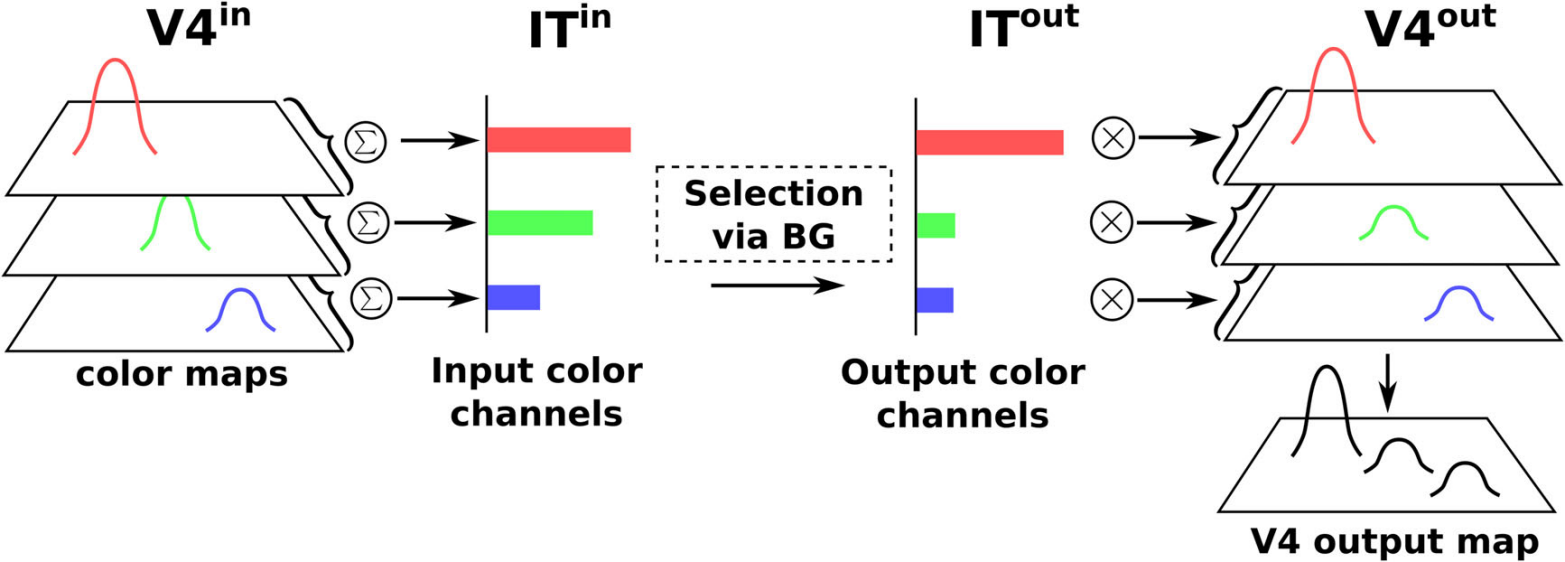
**Spatial-color transformation**.

Thus, the BG selection occurring in the V4|IT-BG-Th loop deals with non-spatial color information only. Then these channels are transformed back into retinotopic maps (cf. Figure 4) and the resulting map (V4 output map) is then projected to SCi. Activity fed to the channels is computed as follows:

(4) $$\begin{array}{l} IT_{c}^{out}=W_{IT^{in}}^{IT^{out}}.IT_{c}^{in}\times (1-W_{SG_{inhib}}.SG_{inhib})\\ \;+W_{Th^{IT}}^{IT^{out}}.Th_{c}^{IT} \end{array}$$

(5) $$\begin{array}{l} Th_{c}^{IT}=IT_{c}^{out}\times (W_{IT^{out}}^{Th^{IT}}+W_{GPi}^{Th^{IT}}\times (1-\frac{GPi|SNr_{c}^{color}}{GPi|SNr_{rest}}))\\ \;-W_{TRN^{IT}}^{Th^{IT}}.TRN^{IT}+I_{Th} \end{array}$$

with *c* ∈ [*red, green, blue*], *IT*^*in*^_*c*_ the visual input channel for color *c, IT*^*out*^_*c*_ the activity of the *IT* layer connected with *Th*^*IT*^ and *SG*_*inhib*_ the ascending inhibition from saccade generators. Thalamic activity depends on *IT*^*out*^_*c*_ and on BG output nuclei *GPi|SNr*_*color*_. The BG output thus gates a part of the transmission between *IT*^*out*^ and *Th*^*IT*^ with a modulating inhibition. *TRN*^*IT*^ is the activity of the globally inhibiting inputs from the thalamic reticular nucleus and *I*_*Th*_ a constant tonic activity. *IT*^*in*^_*c*_ is fed to the reinforcement learning module for the color (*AC*_*color*_). Details of the reinforcement learning are given below (Section 2.2.3). Then, the resulting channels along with *IT*^*out*^_*c*_ are given as inputs to the BG. For full details about Th and BG model see Girard et al. (2008).

**Figure 5:**
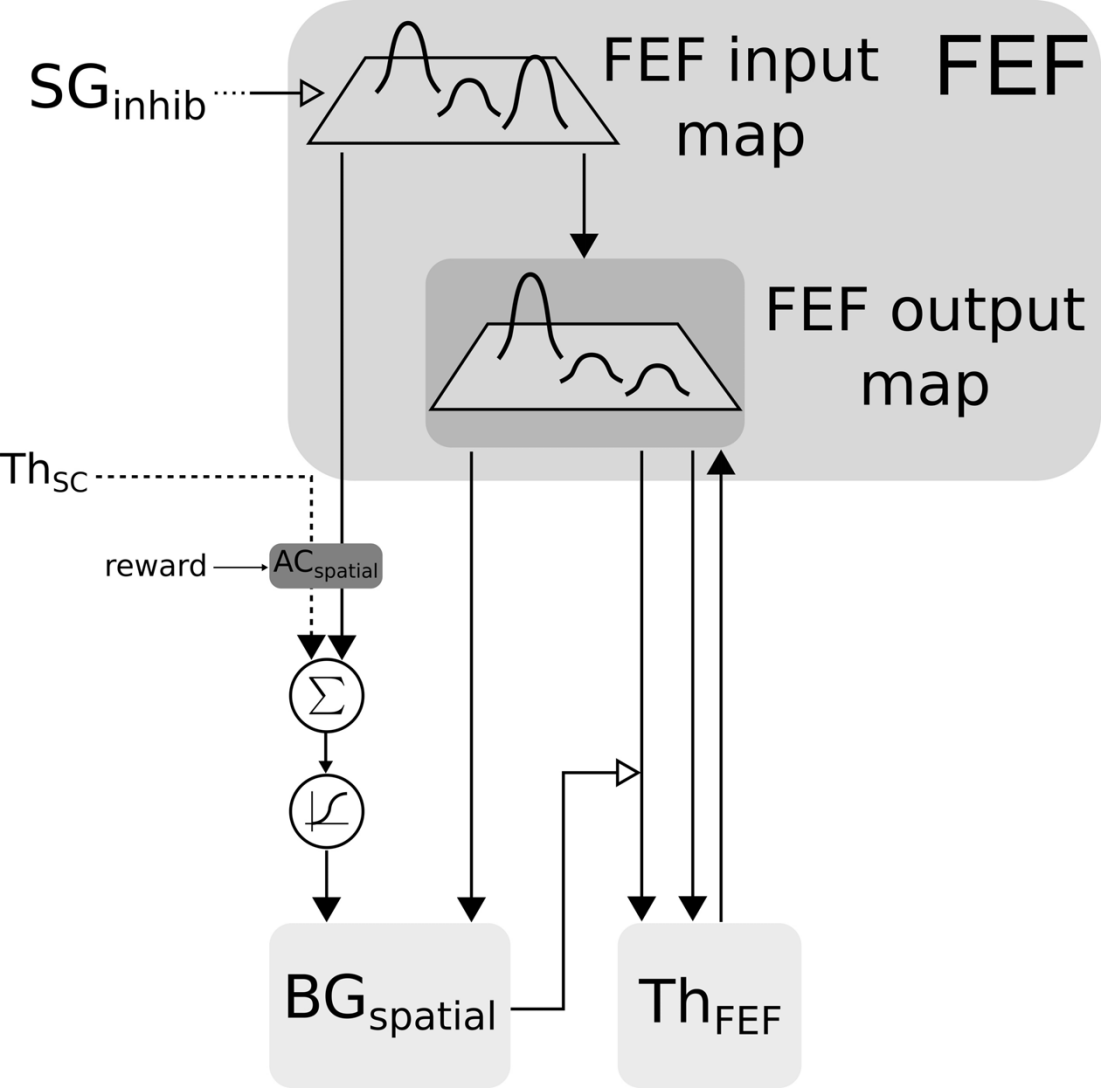
**Closed loop selection-amplification of spatial FEF map**. Black arrow heads are exitatory connexions, empty arrow heads are inhibitory connexions.

Spatial information is processed by two cooperating loops. In the cortical FEF-BG-Th loop, FEF receives visual information in its input map with a long latency (91 ms). This map is then fed to the selection loop (cf. Figure 5) and the resulting activity is computed as follows:

(6) $$\begin{array}{l} FEF_{i,j}^{out}=W_{FEF^{in}}^{FEF^{out}}.FEF_{i,j}^{in}\times (1-W_{SG_{inhib}}.SG_{inhib})\\ \;+W_{Th^{FEF}}^{FEF^{out}}.Th_{i,j}^{FEF} \end{array}$$

(7) $$\begin{array}{l} Th_{i,j}^{FEF}=FEF_{i,j}^{out}\times \left(W_{FEF^{out}}^{Th^{FEF}}+W_{GPi|SNr}^{Th^{FEF}}\times (1-\frac{GPi|SNr_{i,j}^{spatial}}{GPi|SNr_{rest}})\right)\\ \;-W_{TRN^{FEF}}^{Th^{FEF}}.TRN^{FEF}+I_{Th} \end{array}$$

with (*i, j*) ∈ [0, *n*]^2^, *FEF*^*in*^ the visual input and *FEF*^*out*^ the activity of the *FEF* layer connected with *Th*^*FEF*^.

The two maps (*Th*_*SC*_ and *FEF*_*in*_) are concatenated and fed to the reinforcement learning module. We decided to keep both maps concatenated in order to preserve the full learning capabilities and then to merge back the resulting weighted maps at the *BG*_*spatial*_ input level before BG selection.

The merge is done by summing and passing these maps through a sigmoid $\left(f(x)=\frac{1}{1+e^{15.(0.95\;-\;x)}}\right)$, inducing a non-linearity and a minimal salience threshold. Similarly to the color loop, the resulting map along with *FEF*^*in*^ are given as inputs to the BG.

**Figure 6:**
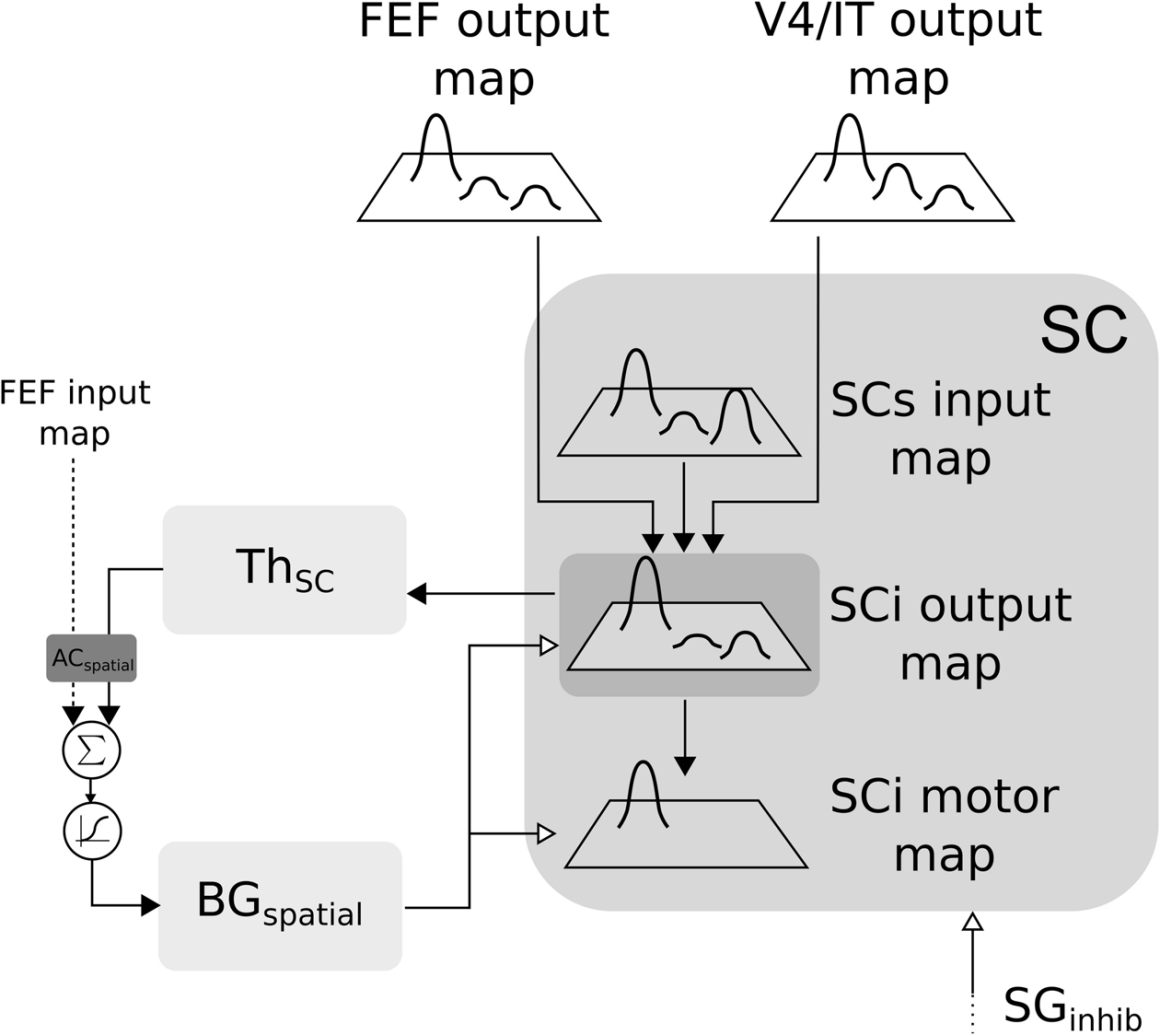
**Closed loop selection-amplification of spatial SC map**. Black arrow heads are exitatory connexions, empty arrow heads are inhibitory connexions.

In the SC-Th-BG loop, SCi receives inputs from V4|IT, FEF and retina (via SCs). These inputs are weighted summed and fed to the selection loop (cf. Figure 6). As stated previously the *BG*_*spatial*_ module is the same than in the FEF-BG-Th loop. The resulting activity is computed as follow:

(8) $$\begin{array}{l} SCi_{i,j}^{out}=\left[\left(W_{SCs}^{SCi}.SCs_{i,j}+W_{FEF}^{SCi}.FEF_{i,j}^{out}+W_{V4|IT}^{SCi}.V4|IT_{i,j}^{out}\right)\right]\\ \;\times \left[W_{SCi^{in}}^{SCi}+W_{BG_{amp}}^{SCi}\times (1-\frac{SNr_{i,j}}{SNr_{rest}})\right]\\ \;\times (1-W_{SG_{inhib}}SG_{inhib}) \end{array}$$

(9) $$\begin{array}{l} Th_{i,j}^{SC}=SCi_{i,j}^{out}\times \left(W_{SCi^{out}}^{Th^{SCi}}+W_{GPi|SNr}^{Th^{SCi}}\times (1-\frac{GPi|SNr_{i,j}^{spatial}}{GPi|SNr_{rest}})\right)\\ \;-W_{TRN^{SCi}}^{Th^{SCi}}.TRN^{SCi}+I_{Th} \end{array}$$

with (*i, j*) ∈ [0, *n*]^2^, *SCs* the visual input from the superficial layer of SC, *GPi|SNr* the inhibition from the output nucleus of BG projecting to SC.

#### 2.2.2. Basal ganglia

**Figure 7:**
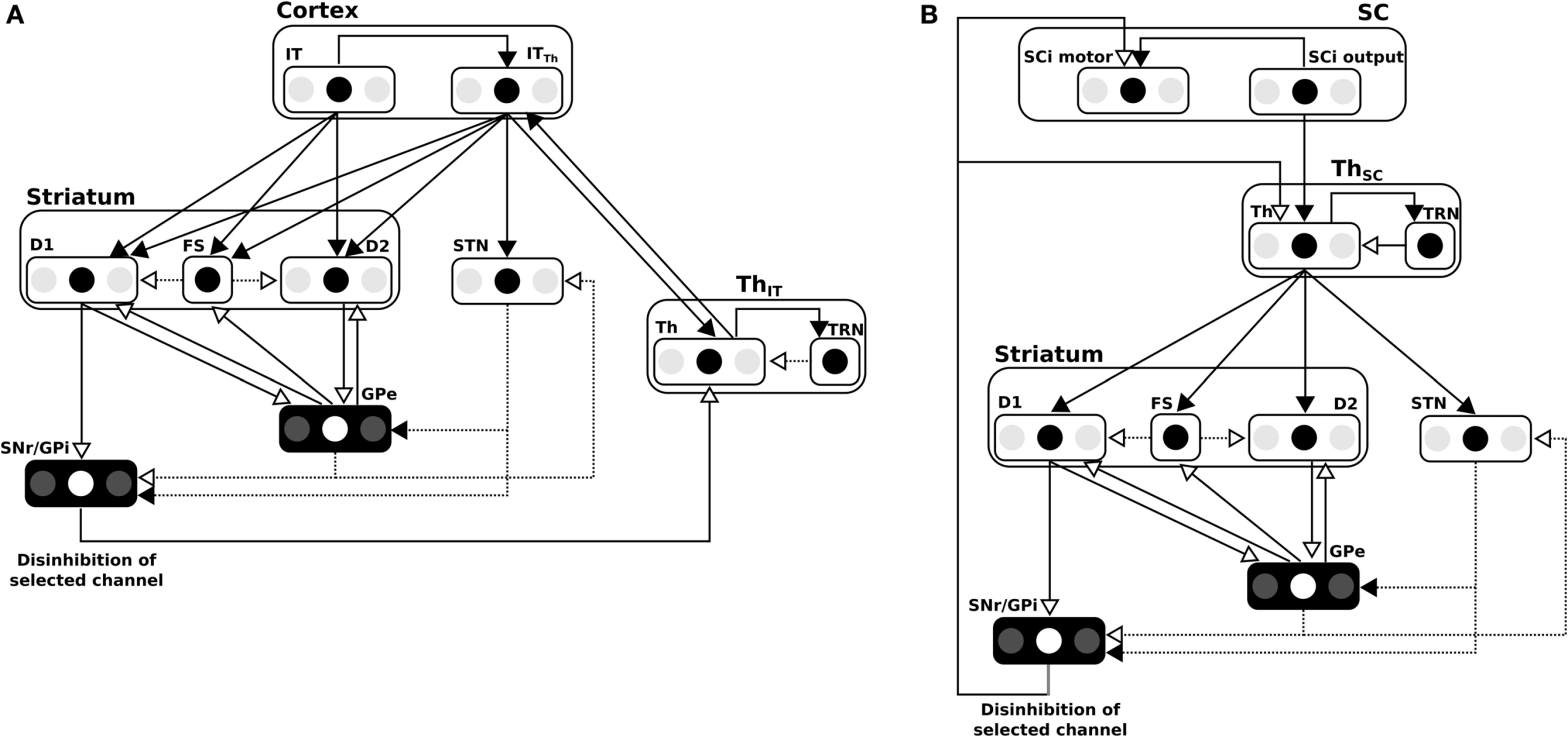
**(A)** Details of the BG model in the cortical loop (here, IT-BG-Th is shown but an identical structure is used for FEF-BG-Th). Only 3 channels are represented, the middle one being the most salient. SNr/GPi and GPe are color inverted as channels activity in these structures are opposed (middle channel which is the most activated in input, is the weakest in these structures). Thalamus structure (Th) is composed of a ventral anterior nucleus and of reticular nucleus (TRN) which constitute a population without segregated channels. Striatum is composed of D1 and D2 types of dopaminergic neurons and of a population of fast discharge inter-neurons (FS). Filled arrow heads are exitatory connexions and empty arrow heads are inhibitory. Filled lines represents one-to-one connexions and dotted lines represents one-to-all connexions. Adapted from Girard et al. (2008). **(B)** Details of the BG model in the subcortical loop (SC-Th-BG). Same model than in **(A)** except for the position of the Thalamus.

The BG model used here was first described in Girard et al. (2008) and is depicted in Figure 7A for cortical loops. Notice that for the subcortical loop the connectivity is slightly different for the position of the Thalamus (cf. Figure 7B).

The parameters of the BG circuit involved in the spatial loop have been adapted so as to cope with the selection of 630 channels (see Supplemental Data).

The *n* × *n* inputs from the spatial maps (here with *n* = 70 for each hemifields) converge on the *m* × *m* inputs (here *m* = 18 for each hemifield) by the Gaussian Pyramids method. Input map size is reduced by first convolving it with a 5 × 5 gaussian kernel:

(10) $$BG_{i,j}^{spatial}={\left(In*G\right)}_{i,j}$$

with *In* the input map, *G* the normalized gaussian kernel and (*i, j*) ∈ [0, *n*]^2^. Then it is 2 × 2 binned in order to divide dimensions by 2.

This operation is repeated 2 times in order to reduce the input map by a factor 16. The opposite operation is computed to upscale the output activity of BG in order to match the projection toward other structures. This dimensionality reduction is inspired by the anatomy of cortico-striatal connections (Zheng and Wilson, 2002).

As seen on Figure 4, the color BG circuit receives the sums of the activity of the color maps, and thus operates selection among three channels:

(11) $$BG_{c}^{color}=W_{IT_{c}}^{BG}\sum_{i,j}IT_{c_{i,j}}$$

with *W*^*BG*^_*IT*_*c*__ a normalization constant. The output of the same circuit thus affects the whole color maps in the following manner:

(12) $$V4_{c_{i,j}}^{out}={\hat{V4}}_{c_{i,j}}.IT_{c}^{out}$$

with ${\hat{V4}}_{c_{i,j}}$ the normalized activity of the input map for color *c*, ${\hat{V4}}_{c_{i,j}}$ = *V*4^*in*^_*c*_*i, j*__/*max*(*V*4^*in*^_*c*_) and *IT*^*out*^_*c*_ the output activity for a whole channel *c*.

#### 2.2.3. Actor critic

The input to the BG circuits is biased by reward using the classical “Actor-Critic” TD(λ) learning algorithm (Sutton, 1988; Montague et al., 1996; Sutton and Barto, 1998).

TD-error δ is computed according to

(13) $$\begin{array}{l} \;\delta =R_{t}+(\gamma \times V_{t})-V_{t-1}\\ \text{with}\\ \;V_{t}=W_{Critic}\cdot Input_{t} \end{array}$$

*R*_*t*_ being the reward at time *t, V*_*t*_ the estimated value function, *W*_*Critic*_ the learned weights of the Critic, *Input*_*t*_ the input matrix (spatial or color) and γ the discount factor.

Critic's weights are then updated using eligibility traces *E*_*Critic*_:

(14) $$\begin{array}{c} \begin{array}{l} W_{Critic}\leftarrow W_{Critic}+\eta \times \delta \times E_{Critic}\\ \text{ with}\\ E_{Critic}\leftarrow \lambda \times E_{Critic}+Input_{t-1} \end{array} \end{array}$$

η being the learning rate and λ the “forgetting” factor of eligibility traces. The size of the Critic's weights vector is *N*, the same as *Input* so here connexions are “all-to-one” type. Actions vector (weighted inputs) is computed as following:

(15) $$A_{t}=W_{Actor}\cdot Input_{t}$$

and Actor's weights are computed as following:

(16) $$\begin{array}{c} \begin{array}{l} \;W_{Actor}\leftarrow W_{Actor}+\eta \times \delta \times E_{Actor}\\ \text{ with}\\ E_{Actor}\leftarrow \alpha \times E_{Actor}+Input_{t-1}⊗{A^{\prime }}_{t-1}\\ \text{ and}\\ \;{A^{\prime }}_{t-1}=GPi_{t-1} \end{array} \end{array}$$

Actor's weights matrix is of size *N* × *N* so here, connexion are “all-to-all” type.

**Figure 8:**
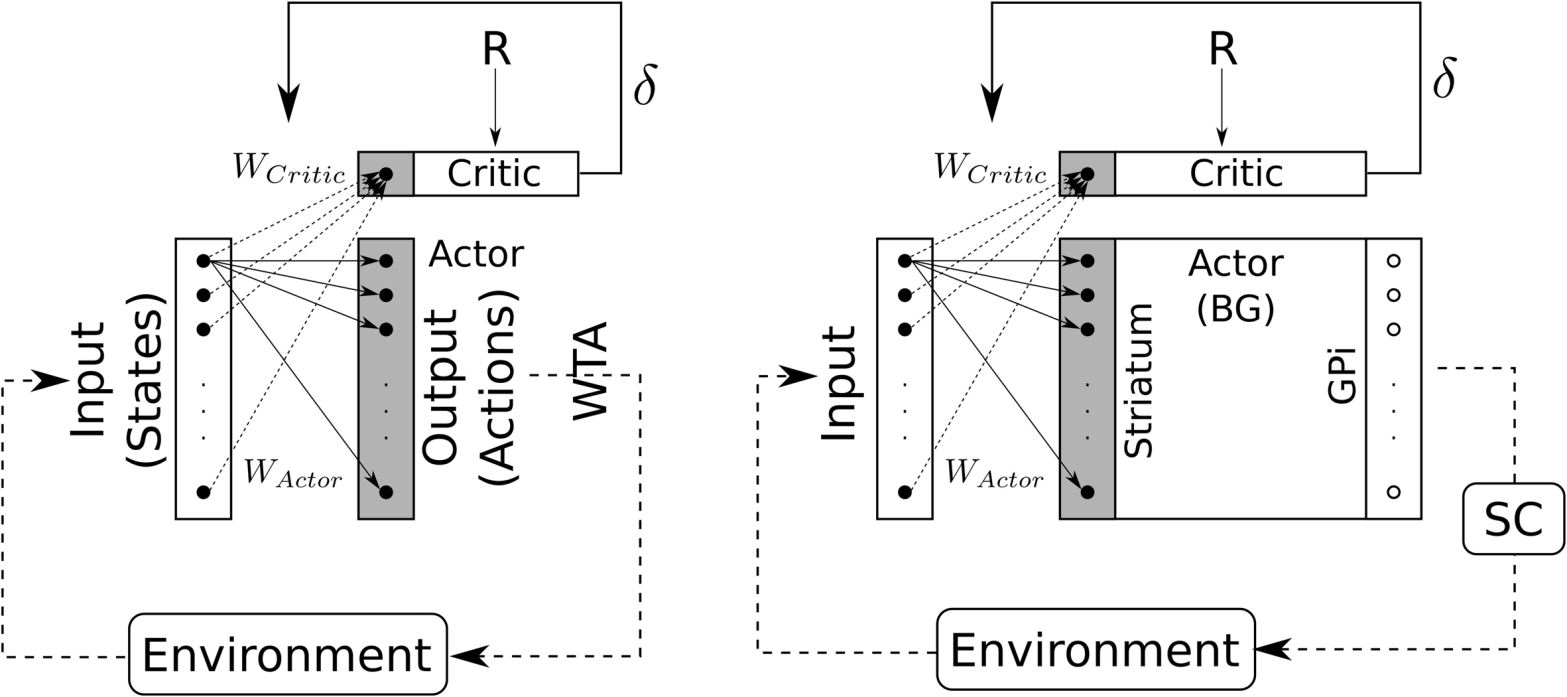
**Schematic representation of the Actor-Critic reinforcement learning algorithm**. Left: classical Actor-Critic, involving a winner-takes-all (WTA) selection mechanism. Right: Actor-Critic with BG as selection mechanism.

Compared to classical reinforcement learning (cf. Figure 8, left) we can see that “States” are inputs to be selected and “Actions” are weighted inputs. Here, the BG compute a selection of these weighted inputs – thus playing the role of the “winner-takes-all” (cf. Figure 8, right) – and then disinhibit some structure (i.e., SC) which eventually will trigger a real action.

Actor's weights are initialized to an identity matrix in order to allow for an initial “standard” behavior (direct unweighted projection). A minimum value for Actor's weights diagonal has been implemented (*W*_*Actor*_*min*__ = 0.6) in order to prevent the system to from losing the ability to trigger saccades. Critics weights are initialized to a random matrix with values ∈ [0, 0.01].

The exploration, which is important for RL convergence, is caused here by a perceptual noise only. This perceptual noise is implemented in the following manner: one input has an amplitude of 1 and the other of 0.95. This 5% difference is sufficient for the system to select the most “intense” input, before learning adds its own biases to the selection, and is randomly alternated between cues in order to ensure the absence of a systematic bias.

#### 2.2.4. Spatio-temporal transformation

**Figure 9:**
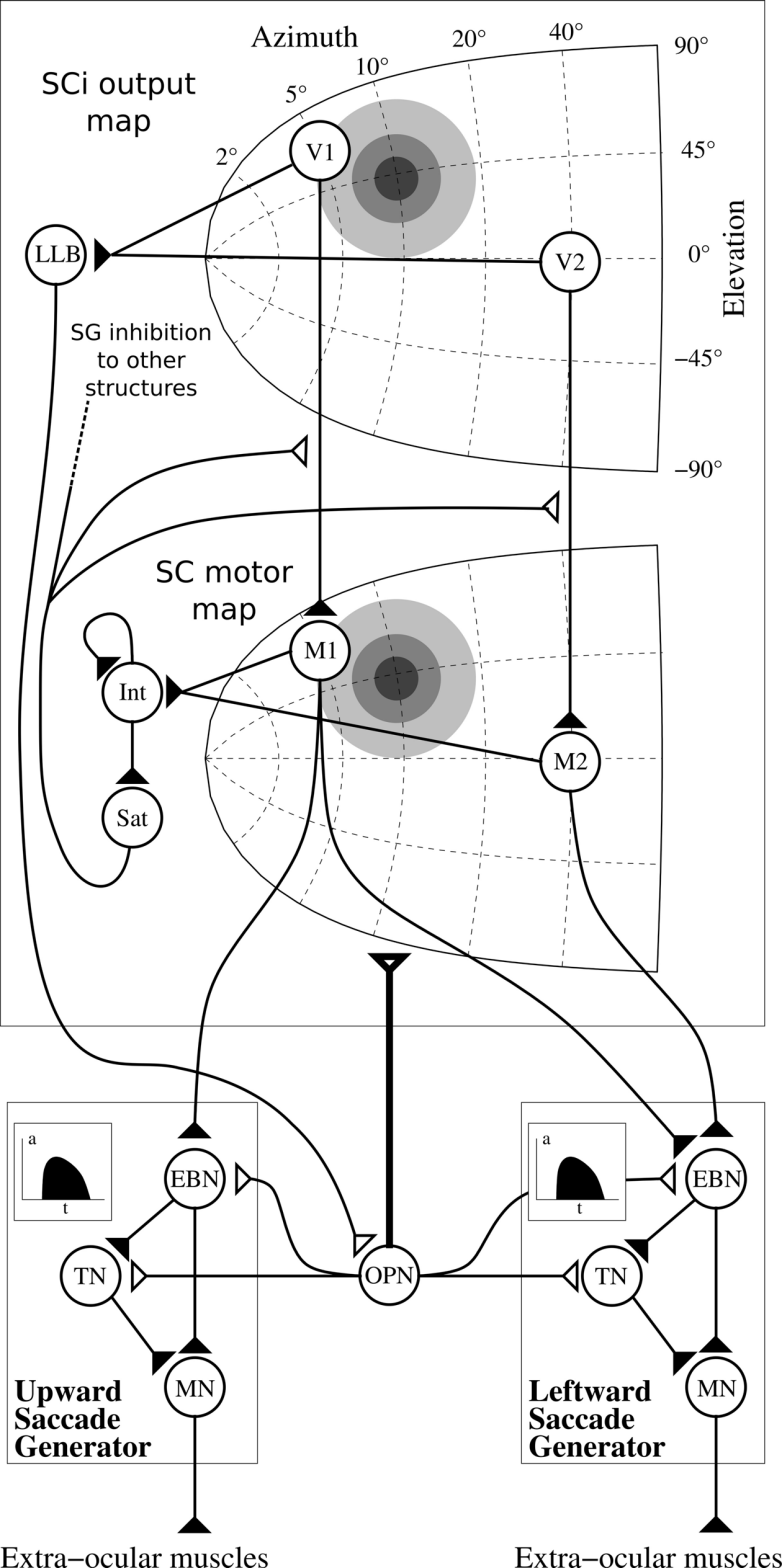
**Architecture of the motor layer of SC**. Only one colliculus (right hemifield) and two SBG are represented (without cross projections) along with two neurons by map (V1 and V2 in the SCi output map, M1 and M2 in the motor map). Gray discs represents gaussian activity produced by a visual target (coordinate (10°, 10°), thus *R* = 10°, θ = 45°), insets in the saccade generator represent the temporal coding in EBNs generated to control muscles. Filled triangles are for excitatory connexions, empty triangles are for inhibitory connexions. Bold connexion affect the whole map. Adapted from Tabareau et al. (2007).

In order to compute the so-called “spatio-temporal transformation” (STT) required to convert a spatially coded target into a saccade burst generators (SBGs) temporal sequence, we used the model first described in Tabareau et al. (2007) (cf. Figure 9). This model includes a visual map (SCi output map described above) and a motor map (SC motor map) with a log-complex mapping along with colliculi gluing mechanism. The motor layer is projected to the saccade generators and both are controlled by a strong inhibition from omnipause neuron (OPN).

We can notice than we slightly modified the “integrating-saturating” mechanism (*Int* and *Sat* in Figure 9). This mechanism no longer inhibits the whole motor map in a subtractive manner, but now modulates the visual map to motor map projection in a multiplicative manner:

(17) $$\begin{array}{l} \begin{array}{l} I_{i,j}^{motor}=SCi_{i,j}^{out}\times (1-W_{BG_{inhib}}^{SCi}\times SNr_{i,j})\\ \;\times (1-W_{Sat}^{Mot}.Sat)-W_{OPN}^{Motor}.OPN \end{array} \end{array}$$

with *I*^*motor*^ the input activity of motor layer, *SCi*^*out*^ the activity of *SCi*^*out*^ map described in section 2.2.1, *OPN* the output activity of the OPN and (*i, j*) ∈ [0, *n*]^2^.

This modification has the advantage of generating more realistic burst activities, more similar to the gamma functions used in van Opstal and Goossens (2008).

Notice that *Sat* is used as the ascending inhibitory signal *SG*_*inhib*_ in other structures, which signals the execution of a saccade (Sommer and Wurtz, 2002).

#### 2.2.5. Model parameterization

The parameters of the model were hand-tuned, these tuning operations were performed, as much as possible, by considering the various subsystems (BG models, generation of the motor command, convergence of the inputs on the SC, and reinforcement learning) in isolation and enforcing their correct operation.

The parameters of the spatial BG loop had to be modified compared to the initial parameterization of (Girard et al., 2008), as the number of competing channels is much higher. This drastically affects the effects of diffuse projections, like those of the STN on the GPe and GPi. When 630 channels are exciting the GPi, rather than 6, the strength of this excitation has to be reduced, so as to avoid saturating the GPi neurons, and so as to allow one-to-one inhibitions from the Striatum to be strong enough to conteract excitation and thus allow selection. These modifications were made as follows: the BG model was isolated from the rest of the system, and provided with 2D Gaussian inputs similar to those used in the tasks, with varied amplitudes. The parameters were adjusted until the selection of a single target with an amplitude between 0.6 and 1 was restored. Finer adjustment were then made so that one or two distractors of inferior amplitudes would not disturb the selection process, and that the simultaneous selection of multiple targets occurred only when they have very close amplitudes.

The parameters of the motor layers of the SC, and of the saccade generators, which operate the spatio-temporal transformation, were almost identical to those of (Tabareau et al., 2007), except slight modifications in the integration rate of the saturating mechanism, so as to adjust the duration of the motor bursts to more realistic values.

The parameters adjusting the strength of the contributions of all the different maps to the final *SCi* layer were adjusted so that: (1) imposing an input from the spatial system only, or from the color one only, would generate the corresponding saccade, and (2) simultaneously imposing a given target position in the spatial system and another one in the color system, would result in an averaging saccade.

Finally, the parameters driving the temporal integration of reward in the learning modules –namely the discount factors γ and the eligibility trace λ– had to be large enough, so that learning could occur despite the relatively long delay between the appearance of a target and the effective reward delivery (≈500 *ms*). The learning rates were adjusted so that the learning would converge to the best possible level of performance in approximately 20–25 sessions. The relative difference between η_*spatial*_ and η_*color*_ has to be considered in the light of: 1) the huge difference in the number of input weights to be adjusted in each system (1587600 in the spatial domain vs. 9 in the color one), and (2) the different extent of the input stimulations corresponding to one target (a 2D Gaussian input spreading over a hunded of channels in the spatial domain vs. one single channel in the color domain).

### 2.3. Simulated tasks

**Figure 10:**
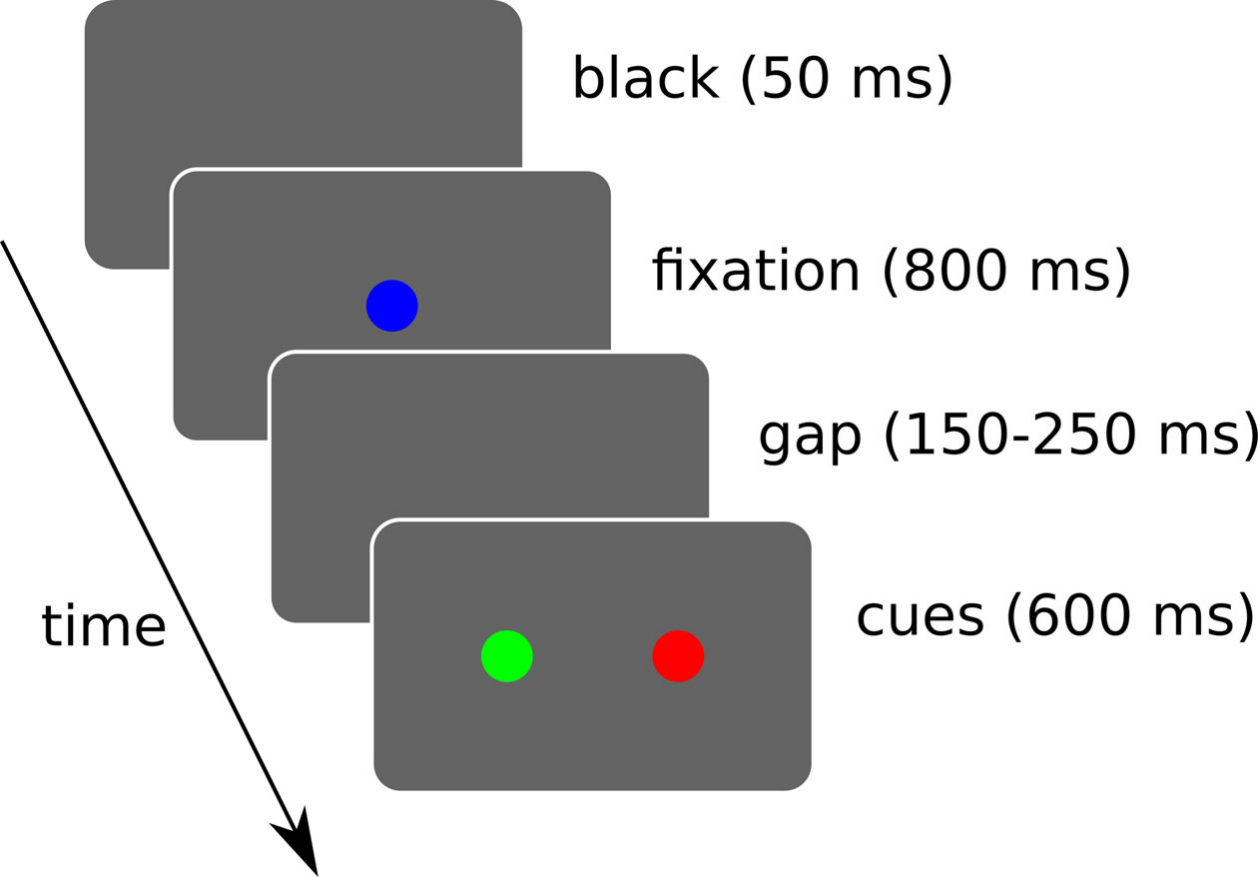
**Simulated sequence of visual stimuli**. A black screen of 50 ms is followed by a fixation cue for 800 ms. Then a random gap time (between 150 and 250 ms) is followed by the two cues. The cues are displayed for a maximum of 600 ms and loops back. During this interval, if a saccade of sufficient amplitude (>2.5° from the center) is detected, the trial ends and loops back. Rewards are given when the trial ends, which may be triggered by the timer or a saccade depending on the task.

We simulated 3 target selection tasks where the system has to trigger a saccade toward one of the two displayed cues (cf. Figure 10).

A “spatial task” is aimed at verifying its ability to learn to choose a target based on spatial information only. A “color task” for color information only. And a “conjunction task” to study interactions between these two. 10 runs were done and each experimental run is composed of 40 sessions of 12 trials.

## 3. Results

### 3.1. Spatial task

In the spatial task, the rewarded cue only depends on its position on the visual field. So the system has to learn to ignore the color information and to favor the spatial one.

**Figure 11:**
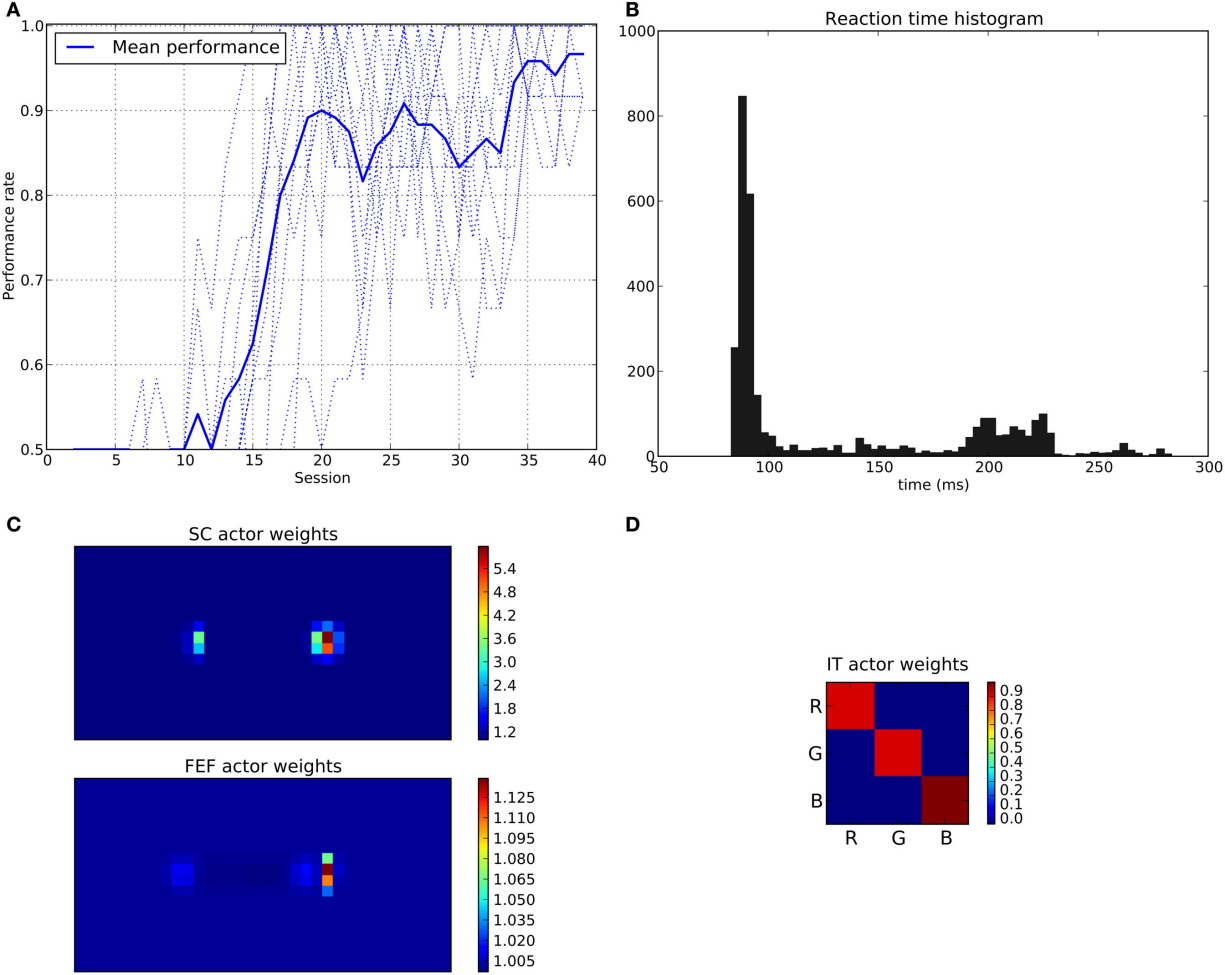
**Results of the spatial task**. The rewarded cue is the right one regardless its color. **(A)** Performance across sessions (bold line is the mean performance of the 10 runs represented with dotted lines). **(B)** Distribution of saccadic reaction time (SRT) for the whole experiment. **(C)** Learned weights (averaged over 10 runs) for the Actor part of the spatial loop; for readability reasons, the multidimensional weight matrix has been projected on the output: it represents, for each unit, the sum of the input weights coming from the whole map, for the SC (top) and the FEF (bottom), note also the different intensity scale between SC and FEF. **(D)** Learned weights (averaged over 10 runs) for Actor part of the color loop.

We can see that the model is able to learn the task with a performance reaching ≈90–95% (Figure 11A), this means that it is possible to find a parameterization of the model allowing for a good level of performance after learning

**Figure 12:**
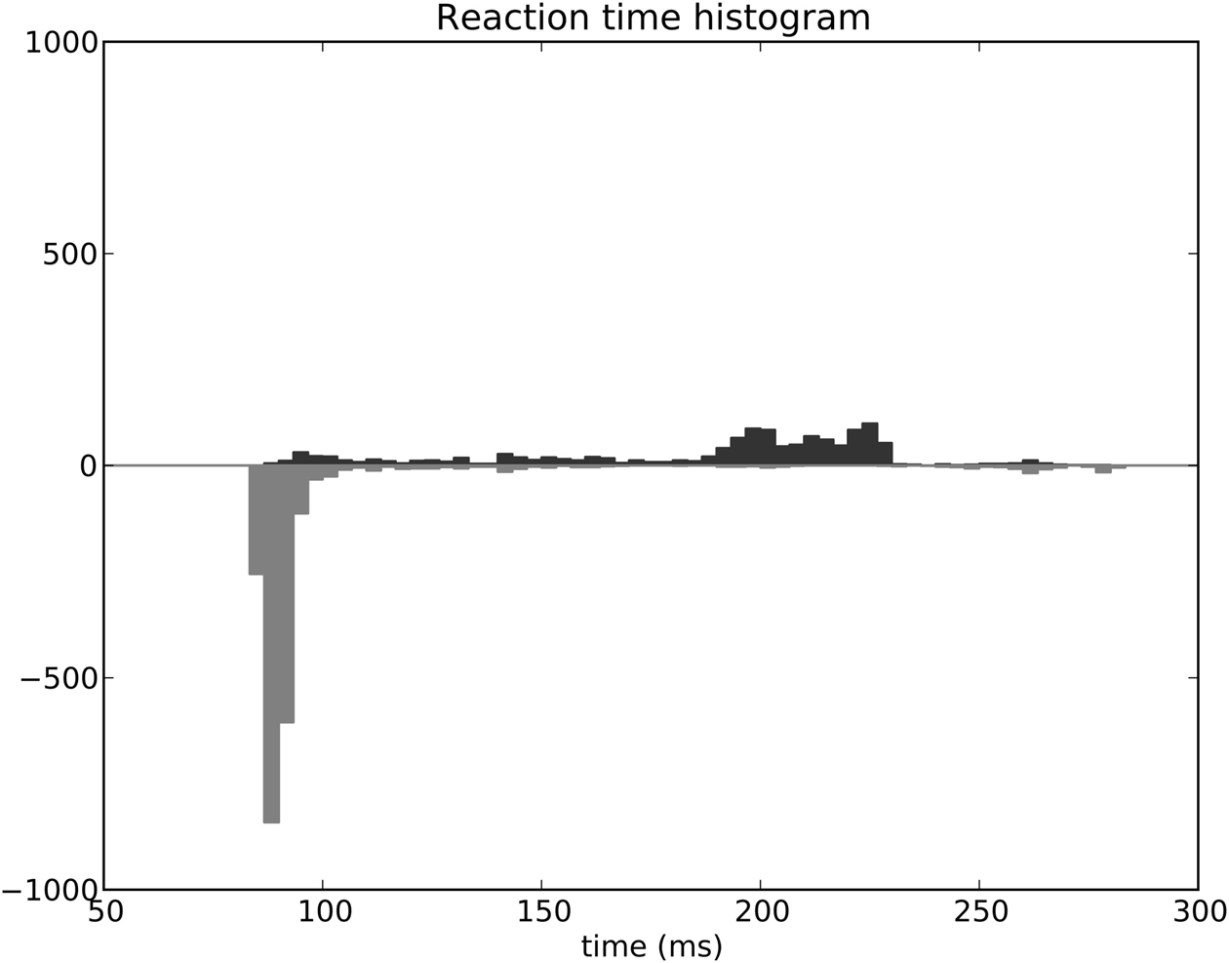
**Spatial task reaction time histogram with separated first half of the experiment (top) and second half (bottom)**.

The distribution of SRT is bimodal, with a very sharp peak of low latency (≈88 ms) and a second bump centered around ≈200 ms (cf. Figure 11B). This behavior is very similar to that of “express saccades” for short latencies and “regular saccades” for longer ones described in Fischer and Weber (1993). Looking at details of the evolution of these SRT, it appears that for the first half of the experiment (first 20 sessions = first 240 trials) saccade latencies mainly fall within the 200 ms mode (cf. Figure 12). These saccades reflect the baseline timings of the system without any selection bias from learning.

For the second half of the experiment (where performance is close to 90%), saccade latencies fall within the 88 ms mode.

Associated weights for the color loop (Figure 11D) indicates that the colors of targets (red or green) have not been learned: they have similar weights values of ≈0.8 in the diagonal.

In contrast, the weights of the spatial loop (Figure 11C) show a strong bias toward the right target (the rewarded one), especially in the weight map corresponding to the SC (≈5.5, while the FEF ones are around 1.1). These weights causes a strong activity on the spatial loop with a quick disinhibition from the SNr as soon as the direct retina-to-SC signal appears. Then, activity is transmitted to the motor layer even before visual information reaches the cortical visual areas and rapidly triggers a saccade. This kind of saccade thus differs from “standard” ones as they only rely on the direct retina-to-SC pathway. Indeed, before learning, the retina-to-SC input is not sufficient to trigger a saccade alone in our model and needs either FEF or V4|IT input, thus explaining the longer SRT.

**Figure 13:**
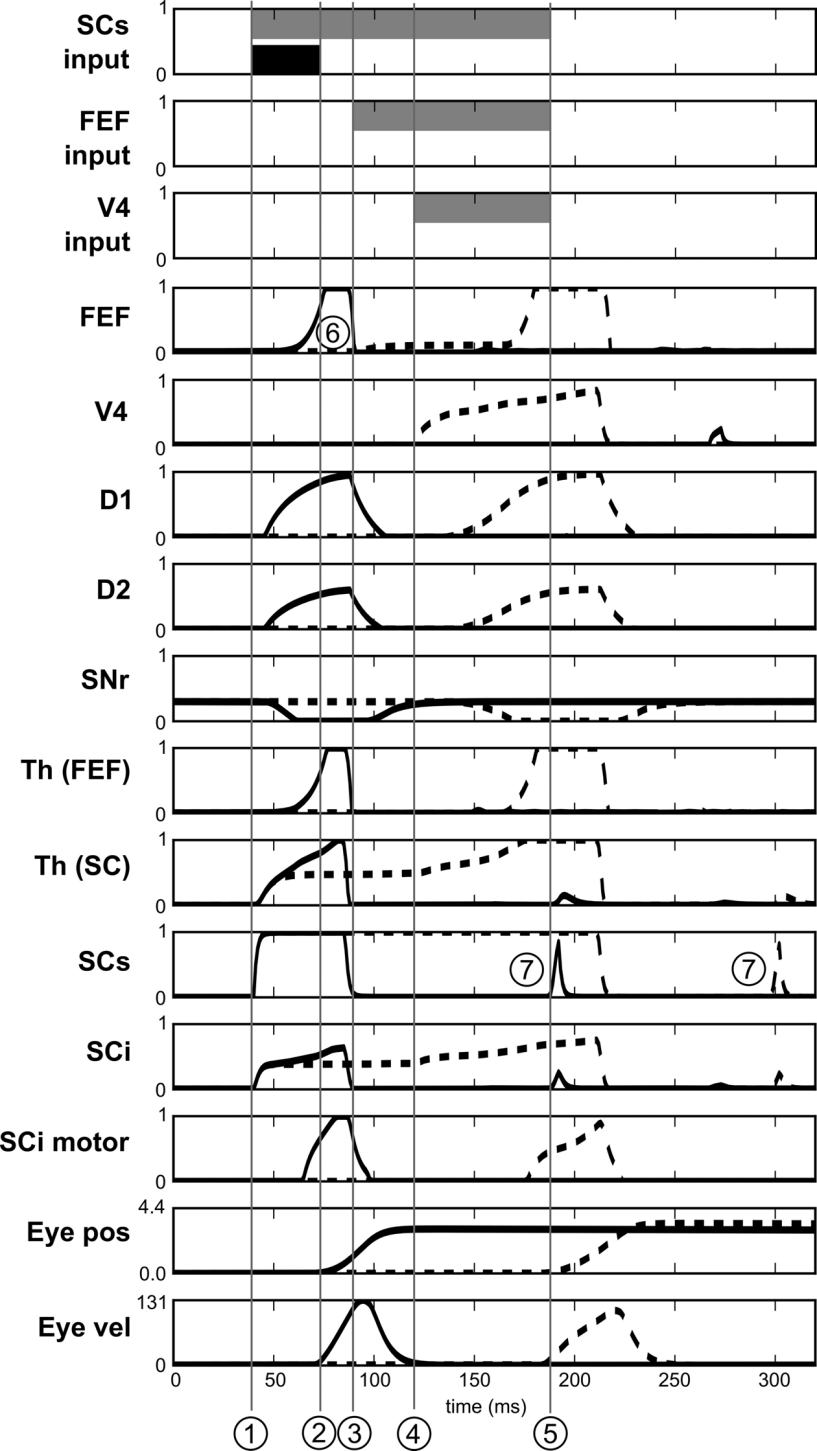
**Activities of different neurons of the target channel in the spatial task**. Target appears at *t* = 0. Dashed line: before leaning. Solid line: after learning. SCs input, FEF input and V4 input represent the presence of the visual cue in the receptive field before learning (gray) and after learning (black). 1: Visual activity reaching SCs. 2: Beginning of the express saccade (after learning). 3: Visual activity reaching FEF. 4: Visual activity reaching V4|IT. 5: Beginning of the saccade before learning. 6: Indirect short latency activity in FEF provoked by SC activity. 7: Small burst of post-saccadic visual activity provoked by the end of inhibition from *SG*_inhib_.

If we look at the details of neural activity in normal and express saccades (Figure 13), what appears for the spatial task (after learning) is that direct retinal input induces activity in the spatial loop, which is quickly dis-inhibited by the BG (thanks to the strong weights) and activates the SCi motor map. Moreover, as the same BG module is shared between the subcortical and the cortical loops, this dis-inhibition also affects the cortical loop and thus induces activity in FEF before visual information reaches it. This induced activity depends in facts on the baseline level of the Thalamus and is a prediction of the model due to our choice of a single shared spatial BG module. The activity in the SC causes a disinhibition in the spatial BG circuit, which then disinhibits also the thalamo-FEF loop. As this loop is auto-excitatory and as the thalamus has a baseline activity, this trigger a resonance between Cortex and Thalamus. Thus the observed short latency activity in FEF is not caused directly by visual input but indirectly by subcortical visual activity.

Yet, express saccades depend only on the SC loop and FEF only has a marginal impact on it. Nevertheless, simulations with a FEF inactivation (after learning) extends SRT of ≈15 *ms*, this FEF resonant activity thus contributes to the global behavior.

Notice that Figure 13 also exhibits some very short bursts of post-saccadic visual activity (better seen for SCs but the mechanism is the same for all the structures). These bursts are provoked by the residual retinal activity reaching each visual region due to the latencies, whereas eyes have already moved. This behavior is probably not significant as it may be canceled by a different choice of parameters for *SG*_*inhib*_ for example.

### 3.2. Color task

In the color task, the rewarded cue only depends on its color. So the system has to learn to ignore the spatial information and to favor the color one.

**Figure 14:**
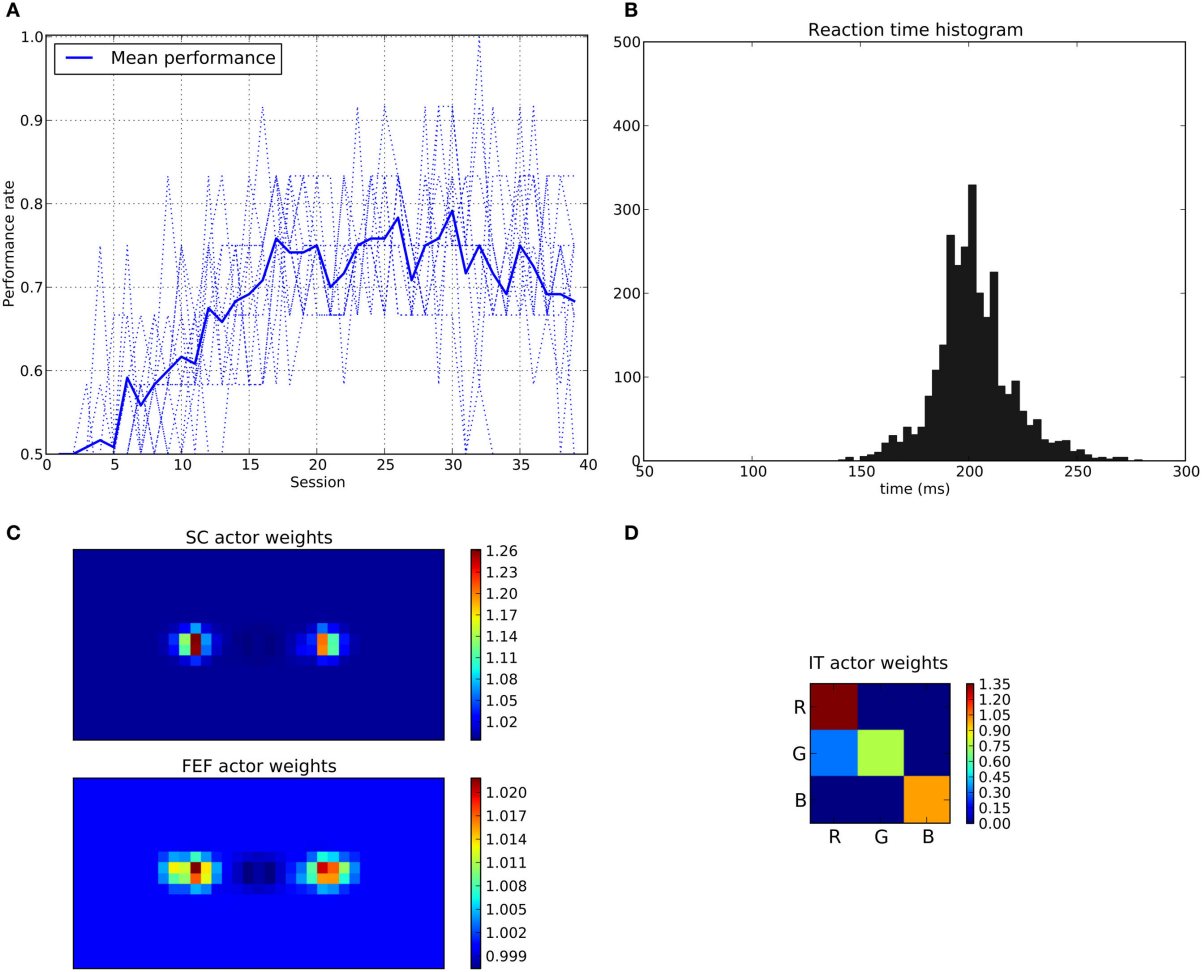
**Results of the color task**. The rewarded cue is the red one regardless its position. **(A–D)** Same as Figure 11.

Here, the average performance only reaches about 75% (cf. Figure 14A), so the system can learn the task but errors are still made at a rather consistent rate. The performance is thus lower that in the spatial task, an effect which is most probably caused by the structure of the BG loops themselves, a point we discuss further in section 4.2.

Color learning is very sensitive to noise in the spatial domain. Indeed, most of the time (≈95%) these errors occur when the distractor (object with the wrong color) is the most intense (“intensity” is imposed to be 1.0 or 0.95 by the perceptual noise). It means that even with learned weights favoring the good color in average (cf. Figure 14D) and spatial ones almost symmetric (cf. Figure 14C, the intensity scale indicates very small variations), the color loop is sometimes unable to impose its choice when a competition occurs between the spatial and the color loop. This is explained by the fact that the subcortical circuit, which operates exclusively on spatial information, can take decisions faster than the color loop. It thus can impose a choice based on spatial information even before the cortical color loop converges to a decision.

The SRT for this task mainly consists on a single mode histogram centered around 200 ms, easily explained by the longer latency of the color loop (122 ms). No reduction of these latencies by learning were to be expected, as no faster pathway operating on colors is available.

### 3.3. Conjunction task

In the conjunction task, the rewarded cue depends on both position and color (e.g., red disk at the right position). When this conjunction is not presented (No conjunction case), the system is rewarded only if the eye position stays within a 2.5° circle around the center (“Good average” behavior).

**Figure 15:**
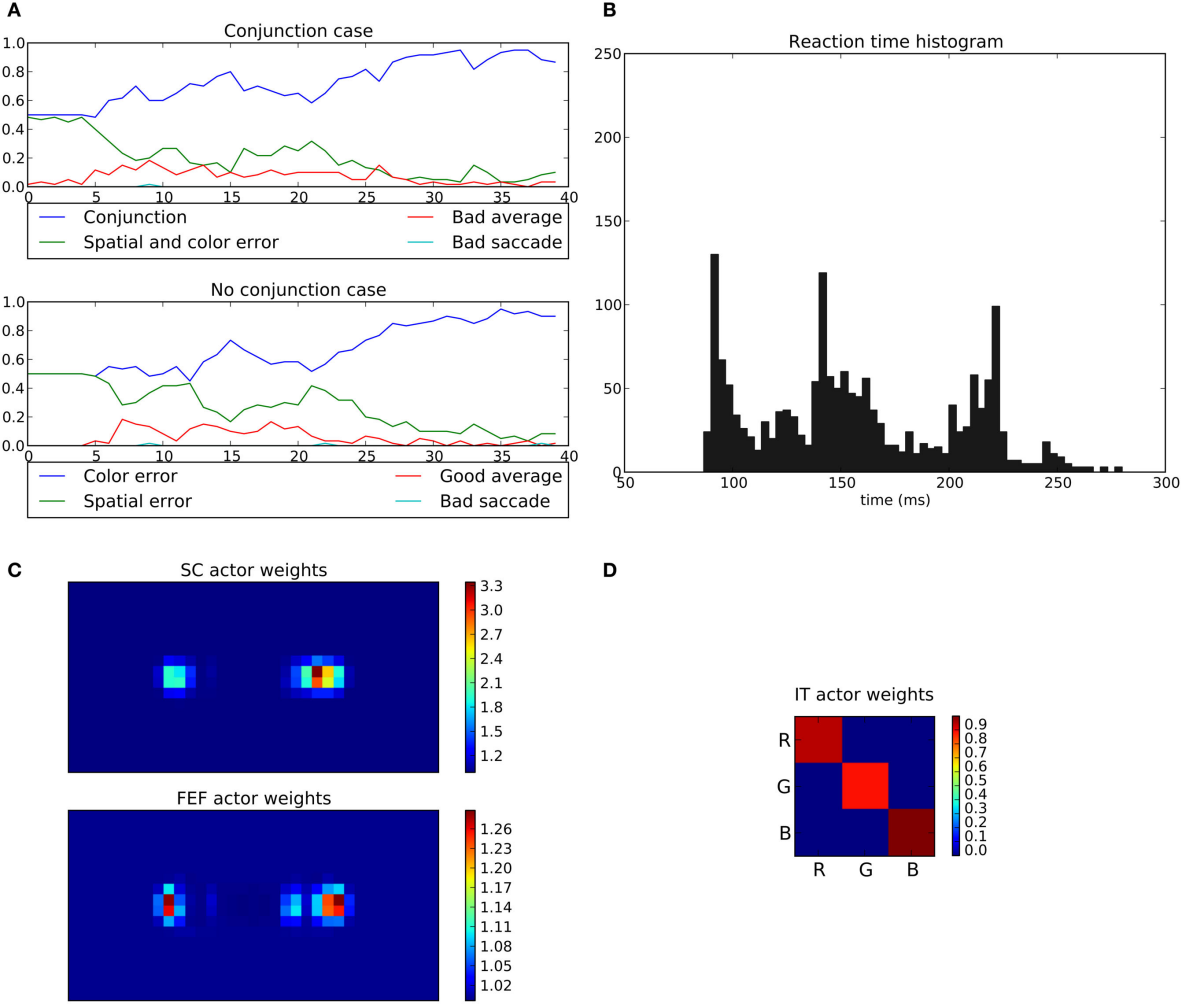
**Results of the conjunction task**. The rewarded cue is the right red one; if not present, reward is given for fixating the center area. **(A)** Average choices, in the conjonction (top) and no conjunction (bottom) case. In the conjunction case, “conjunction” represents the good choice, “spatial and color error” a movement toward the wrong cue, “bad average” an averaging saccade (both targets selected simultaneously) and “bad saccade” (saccades that fall neither within a 2.5 radius from the center or any cue). In the no conjunction case, “good average” is a rewarded saccade keeping the eyes on the fixation point, “spatial” and “color” errors respectively represent movements to the green target on the right and to the red target on the left, and “bad saccade” in any other position (generally between fixation and cue but outside the 2.5 radius). **(B–D)** Same as Figures 11, 14.

Here, the average performance for the conjunction case reaches levels similar to those of the spatial task (around 95%, Figure 15A) but for the “No conjunction case” the rewarded behavior (“Good average”) is rarely performed. We can see that the errors made in this case tend to be mostly “color errors” i.e., a saccade toward the good location but with the wrong color (around 90% of errors at the end of the experiment). “Spatial errors” occurred when a saccade is triggered toward the good color but at the wrong position. However, we can see that at the beginning of the learning and until half of the experiment, the system is still able to produce a small number of “good average” (≈15%). This behavior progressively disappears as the spatial loops learn and become faster, thanks to its subcortical component, making it more difficult for the color loop to select due to its longer latency.

What appears at the end of the experiment is that the behavior of the system is mainly dominated by the spatial loop with almost no weighting from the color loop.

The learned weights correspond well to the task as the right position is favored compared to left one with (cf. Figure 15C) but the red color is only slightly favored compared to green (cf. Figure 15D).

The saccade reaction time is more complicated here. In fact we can see three modes (≈88 *ms*, ≈140 *ms* and ≈220 *ms*). These three modes are in fact explained by the respective latencies imposed for the three pathways, SC (41 ms), FEF (91 ms), and V4|IT (122 ms). Similarly to the spatial task, most of the 88 ms saccades occurred on the second half of the experiment reflecting the specialization toward spatial selection. In fact, saccade latencies shift from the 220 ms mode roughly at the first tier of the experiment, to the 140 ms mode at the second tier and then to the 88 ms mode. This gradual shift of timing thus explains the lack of influence of the color loop, whose pathway latency is of 122 ms. A saccade may be triggered by the spatial loops before feature information even reaches the color loop. Again, this effect is not specific to a given parameterization: the advantage of the spatial decisions, caused by a subcortical circuit with earlier access to information, and thus with faster learning, is structural. It is to be noted that the 140 ms peak did not appear in the spatial task, as the learning is fast enough to allow the system to quickly switch to an “express saccade expert.” This is also explained by the information “redundancy” in our model between SC and FEF, the latter dealing with the same spatial information only with a longer latency. In the conjunction task, this peak appears as the “difficulty” slows down the learning, and thus the shift to an “express saccade expert.”

## 4. Discussion

We described a model of the saccadic system with some very specific structural features:

- the cortico-basal circuits operate in various dimensions (selection based on spatial position, or on target features), with sensory inputs provided with a given latency,
- the subcortico-basal circuit operates on spatial information only, and with a shorter latency,
- all these circuits are subject to reinforcement learning at the level of the input of the BG,

We claim that this structure predicts very specific behaviors, especially in feature-based and space-and-feature-based decisions:

- In the spatial decision task, an ability to switch, from long-latency to short latency saccades (thanks to the learning of the subcortical circuit). An effect experimentally described in Fischer et al. (1984).
- In this task, after the learning of the subcortical shortcut, an early burst of activity in the FEF appears, caused by resonant activity in the spatial circuit. This burst slightly contributes to the reduction of the saccade latency.
- In the color decision task, the concurrently learning subcortical circuit reduces the efficiency of learning, when compared to the spatial task. In normal animals, this effect could be canceled by a external cognitive brake, for example the dlPFC, acting on the subcortical circuit. Thus we predict that this deficit observed in simulation should be observed only in animals with prefrontal cortex deactivation.
- In the conjunction color-and-space-based task, again with the same prefrontal cortex deactivation, space should dominate in the sense that when the cunjunction is not presented, (1) inhibiting the response should be difficult and disappear with learning, (2) the resulting errors should be preferentially directed toward the correct position in space rather than toward the target with the correct color, (3) the saccade latencies should decrease as in the purely spatial task, a clear clue that the subcortical spatial circuit has taken full control of the decisions.

### 4.1. Previous models

Very few models have investigated the operation of multiple basal ganglia circuits in saccadic decision and learning (Girard and Berthoz, 2005), and even fewer took into account the existence of a purely subcortical loop.

The seminal model of Dominey and Arbib (1992, 1995) is quite complete, with memory and sequence learning that we have not yet replicated. Nevertheless, some of its aspects seem now rather outdated. First, their model lacks the subcortical SC-Th-BG loop which is now clearly identified: they only integrated cortical loops. This subcortical loop can operate faster than the cortical circuit and one aim of our work is to explore their interactions. Second, the BG model they used is oversimplified. Indeed it is only based on the direct/indirect interpretation of the BG connectivity, from which they keep the direct pathway only. Consequently, concurrent channels cannot interact in the BG circuitry which make target selection problematic. Their SC motor layer thus requires an *ad hoc* winner-takes-all mechanism, where our more complete BG model solves these problems.

The model proposed in Brown et al. (2004) includes a cortical loop dedicated to saccade strategy selection, and a subcortical loop dedicated to target selection. They also include a working memory mechanisms we have not yet included. Their cortical “strategy” loop explicitly selects whether the target of a saccade will be based on the fixation cue, target position or target feature. Their subcortical loop lacks any thalamic relay and is entirely controlled by the cortical loop, making it unable to learn and make saccades without it. Finally, the details of their BG circuitry suffer from limitations, discussed in details in Girard and Berthoz (2005).

Chambers et al. (2005) proposed a model integrating both the subcortical and cortical pathways without learning capabilities, where a single up-to-date BG model dedicated to location-based selection integrates FEF and SC inputs. Using the various positive feedback loops of this circuitry, they show that manipulating the level of dopamine in their BG model generate reaction time and saccade size modifications reminiscent of Parkinson's disease patient behavior. This model is equivalent to our spatial circuits, and does not explore learning and competition between cortical loops.

The model described in Guthrie et al. (2013) integrates two cortical loops (“cognitive” and “motor”) interacting through different associative structures at both cortical and striatal level. They store in a sub-part of the Striatum all the possible spatial and feature combinations, which could create an obvious combinatorial problem in a realistic model with a full field of view representation and a rich feature space. This model has shown the ability to learn to select targets based on conjunction of information between the two loops but does not include SC and does not specify how the selection in the BG is transformed in a motor command. The associative striatal structure is dependent on the associative cortical one and provides a mean of information transfer between loops. However, the BG architecture used is quite simplified, lacking GPe and GPe-STN connectivity. Finally this model does not include any subcortical loop and thus did not study possible interactions between cortical and subcortical loops.

### 4.2. Spatial dominance

Our results show that the system is able to learn basic behaviors such as the “spatial task” and the “color task.” Moreover, we observed quite different abilities for these tasks. A first difference appeared on the color task performance which only rises to about 75%. This difference can be explained by the very structure of the model where the spatial loop intrinsically dominates the system as it includes the SCi output map and has access to information before the color one. Thus, it can learn before the color loop processes information and, has the last word on selection. This characteristic is confirmed in the “conjunction task” where the system finally learned a “spatial task.” What is quite clear with this architecture is that subcortical spatial choice should prevail when opposed to a color one. This characteristic was also observed in a previous work with a simpler model without the cortical spatial loop (N'Guyen et al., 2010) and seems to be a prediction of this architecture. Such a prediction could be tested on animals with dlPFC inactivation in a task where both a spatial and feature criterion contradict each other as we expect the dlPFC to inhibit impulsive subcortical behavior. This prediction wouldn't be hold for the model proposed by Guthrie et al. (2013) as they explicitly represent conjunction information in the Striatum, and this allows for an experimental discrimination between the two models.

### 4.3. Express saccades

Moreover, another stable outcome of this model relates to the saccade reaction time. We observed what resembles to “express saccades” for the spatial task. These short latency saccades occurred only after a period of learning in our case. This training dependent behavior is in accordance with previous observations on monkeys (and humans) (Fischer et al., 1984; Fischer and Ramsperger, 1986). However it appears that monkeys are also able to trigger some rare and spontaneous express saccades without learning that our model cannot reproduce. This behavior may be viewed as a kind of exploratory one, clearly lacking in our model.

These express saccades are only performed toward learned locations and never toward learned features. This suggests that this behavior is location dependent and not feature dependent, which is in accordance with results in monkeys (Fischer et al., 1984; Schiller and Haushofer, 2005). Indeed, imposed sensory pathways latencies exclude the ability of express saccade for the cortical color loop (122 ms) which easily explains the lack of such saccade in the color task. Therefore, the intrinsic architecture of the model predicts that correct express saccades cannot occur based on feature information. Moreover in our system this spatial dependency is encoded in a retinocentric reference frame and so doesn't depend on the location of target in space which is also in accordance with previous results (Schiller and Haushofer, 2005).

Furthermore it seems that these express saccades are not dependent on FEF as simulations done with FEF inactivation on a learned system, only lengthen them of about 15 ms which seems to be quite in accordance with what was observed in lesion studies (Schiller et al., 1987).

Interestingly, we observed a short latency burst of activity in FEF prior to the execution of the express saccade. This activity is not caused by a direct visual input (it appears before visual input reaches FEF) but by an indirect SC activity causing the a resonating activity in the cortical loop. Although a SC to FEF projection, either direct or through the Thalamus, has been hypothesized (Sommer and Wurtz, 1998; Everling and Munoz, 2000), this induced activity through BG disinhibition seems to be a new prediction of our model.

Notice that the express saccades we obtained could be theoretically shortened even more with a pre-disinhibition of BG which could be viewed as a preparatory activity. Doing so it should be possible to shorten latency by tens of milliseconds maybe explaining the observed range of timings from 70 to 90 ms in living animals. For example a preparatory activity in FEF during the gap period which could either facilitate or even elicit disinhibition of BG (Everling and Munoz, 2000). Whether this pre-disinhibition exists or not remains a question to be answered experimentally. However this phenomenon was not observed in our system and may require some memory capacity that we did not implement.

If we look further at the SRT distributions, what is commonly observed in primates is a bimodal distribution of reaction time for a detection task (only one cue) which can be related to our spatial task. These two modes are in the range of 80–100 ms and 130–160 ms. Moreover, as said before these timings keeps quite unmodified after a FEF lesion but are drastically changed after a SC lesion (Schiller et al., 1987). Our model produces a compatible bimodal distribution but with a longer latency for the second mode which involves the color loop. So it seems that our model doesn't capture the exact mechanism explaining this precise timing.

In contrast, a unimodal distribution is observed in primates for a discrimination task (where the animal has to choose a cue based on a feature) which can be related to our color task. In this case the distribution is wider and in the range of 160–200 ms without express saccades. Once again, this distribution remains unchanged after FEF lesion but is modified after a SC lesion (Schiller et al., 1987). Here the mechanism proposed by our model seems quite consistent with the experimental data.

Unfortunately to the best of our knowledge there is no data on a spatial-feature conjunction task in the literature, but it is to be noted that a similar three peaks distribution was observed in a quite different task where the primate had to choose between two targets (both rewarded) presented with a 50 ms offset (Schiller et al., 2004).

### 4.4. Exploration

Noise is necessary in the system to allow the generation of saccades toward one target among two with similar predicted values, rather than systematically resulting in averaging saccades. While averaging saccades sometimes happen in behaving animals (Ottes et al., 1984) they are quite rare and not as systematic as our model would produce them without perceptual noise. This is because the output of our BG does not represent a probability distribution of possible targets but indeed a direct control that requires a unique choice. Yet, our solution is probably a bit simplistic, a more plausible one would be to produce a selection with more competition between targets such as “race models” (Bundesen, 1987; Ludwig et al., 2007). These mechanisms would most of the time allow a selection of a unique target between two perfectly identical cues. Moreover these mechanisms could also produce an attentional engagement/disengagement behavior which could produce the “gap effect” (Saslow, 1967; Braun and Breitmeyer, 1988) that our model cannot replicate.

### 4.5. Multiple loops

In our model we have chosen to include only one SC-Th-BG loop but McHaffie et al. (2005) have identified at least two (maybe three) different loops involving different layers of the SC.

The first one linking the SC superficial layers (SCs) to the BG via lateral posterior (LP) and pulvinar nuclei of thalamus and ending back to the SC superficial layers (and possibly also deep layers). According to the fact that SCs activity is mainly driven by direct retinal projection, it seems reasonable to think that this loop could be responsible of selection of these retinal inputs. We didn't implement this loop that appeared redundant in our model as we included a SCs to SCi projection but we can imagine a different mechanism with for example a SCs to SCi pathway gated by SNr inhibition.

The second loop—that we implemented in our model—links the SC deep layers (SCi) to BG via intralaminar thalamus nuclei (both caudal and rostral, which represent segregated regions with different type of contact to striatal medium spiny neurons and thus may in fact describe two parallel loops). The deep layers of the SC are known to receive afferent connections from multiple areas (sensory, premotor, motor, but also multisensory…) (May, 2006) thus probably conveying much higher level information. Moreover, as good evidences indicate a SCs to SCi projection (Lee et al., 1997; Isa, 2002), it seems reasonable to think that this loop could be involved in selection of sensory (or high order) targets for orienting behavior as described in this work.

### 4.6. Associative map

The conjunction task clearly requires the ability to select and combine feature and location, but we built our model with the conservative assumption that these different types of information were treated independently by strictly separating feature and spatial loops in the learning stage. We thus stick to the assumption of parallel functionally segregated loops as described in Alexander et al. (1986). Moreover this choice was also driven by anatomical considerations as the TE region of IT seems to projects to the “Visual Striatum” (Middleton and Strick, 1996) while the FEF seems to project to the “Oculomotor Striatum” (Stanton et al., 1988). This architecture should make learning quicker and learning generalization easier (i.e., we can directly learn that a color is rewarded regardless of its position rather than learn each color/location combination). This assumption has also the clear advantage to keep the system simple without the need to learn all possible combinations of features and locations which would causes a problem of combinatorial explosion. But the disadvantage is that the system has no means to directly associate the couple feature/location and can only separately learn both, explaining the relatively poor performances for this task.

We hoped that each loop could learn to select separately and then produce the desired behavior while combined back at SC level. However, with this architecture the only mean to perform the correct behavior (trigger a saccade only if the good cue appears at the good position) is by triggering an average saccade between the two cues in the “no conjunction” case and thus keeping fixation close to the center. In our model, it becomes less and less probable as learning progresses, because the spatial loop becomes quicker than the feature one, thus feature information cannot be included in the decision anymore. Notice that with an external brake (such as inhibition from dlPFC) limiting the expression of express saccades, the task could probably be learned.

Different architectures can be proposed to alleviate this problem in more realistic ways. It is possible to combine all the information at different levels. FEF is known to receive inputs from multiple areas (Schall et al., 1995), being a convergence structure for ventral and dorsal visual stream. In particular in our case, IT (TE) is known to project to FEF (Schall et al., 1995) and we can imagine that FEF already combines spatial and non-spatial information. This combination could occur after feature selection and then explain the observed salience map (Thompson et al., 2001).

Another possibility could be a combination at the Striatum level allowing the possibility to learn combination of inputs as done in Guthrie et al. (2013). The disadvantage is to multiply the size of the input vector as stated above. If we have *N* spatial channels and *M* color channels the input size is *N* × *M* and the all-to-all weight matrix (*N* × *M*)^2^. Even if Guthrie et al. (2013) invoked interesting biological bases, one can question if this kind of combination is a problem in biological systems. The predictions we make about the conjunction case could help deciding based on experimental data, which architecture (separated or merged loops) is correct.

Finally, interaction between loops can also happen at the Thalamus level. Even if FEF and IT loops doesn't share the same Thalamic nuclei (VAmc for IT and MDpl for FEF) this mechanism could still be possible.

## Funding

This research is funded by the HABOT project (Emergence(s) Ville de Paris program).

## Supplementary material

The Supplementary Material for this article can be found online at: http://www.frontiersin.org/journal/10.3389/fncom.2014.00048/abstract

### Conflict of interest statement

The authors declare that the research was conducted in the absence of any commercial or financial relationships that could be construed as a potential conflict of interest.
