# Supplementary material for: Saccade learning with concurrent cortical and subcortical basal ganglia loops
Source: Front Comput Neurosci. 2014 Apr 23;8:48. doi: 10.3389/fncom.2014.00048 (PMC4005946; doi:10.3389/fncom.2014.00048)
Supplement: Supplementary file 1 [file Presentation1.PDF]

Frontiers in Computational Neuroscience

Research Article
2 April 2014

# *Supplementary Material*: Saccade learning with concurrent cortical and subcortical basal ganglia loops

**Steve N'Guyen [1,2*], Charles Thurat [1] and Benoît Girard [1]**

[1] *Institut des Systèmes Intelligents et de Robotique, Université Pierre et Marie Curie-Paris 6, CNRS UMR 7222, Paris, France*
[2] *LPPA, Collège de France, CNRS UMR 7152, Paris, France*

Correspondence*:
Steve N'Guyen
Institut des Systèmes Intelligents et de Robotique, Université Pierre et Marie Curie-Paris 6, CNRS UMR 7222, 4, place Jussieu, 75252 Paris Cedex 05 - France
, steve.nguyen@college-de-france.fr

## SUPPLEMENTAL DATA

### MODEL PARAMETERS

**Supplementary Table 1.** Parameters of the BG model in the spatial loop. The two independent Thalamus modules share the same parameters.

| $N$ | 630 | $\tau$ | $10ms$ | $\tau_{STN}$ | $5ms$ | $\tau_{FS}$ | $5ms$ | $\tau_{FC}$ | $10ms$ |
|---|---|---|---|---|---|---|---|---|---|
| $\tau_{TH}$ | $5ms$ | $\tau_{TRN}$ | $5ms$ | $\gamma$ | 0.2 | $W_{GPe}^{D2}$ | 0.6 | $W_{D2}^{GPe}$ | 0.8 |
| $W_{GPe}^{D1}$ | 0.6 | $W_{D1}^{GPe}$ | 0.0001 | $W_{GPe}^{FS}$ | 0.001 | $W_{FS}^{D1}$ | 0.1 | $W_{FS}^{D2}$ | 0.1 |
| $W_{STN}^{GPe}$ | 0.0003 | $W_{GPe}^{STN}$ | 0.0003 | $W_{GPe}^{GPi}$ | 0.0002 | $W_{STN}^{GPi}$ | 0.0003 | $W_{D1}^{GPi}$ | 0.8 |
| $W_{TRN}^{TH}$ | 0.003 | $W_{TH}^{TRN}$ | 0.003 | $W_{FCtx}^{TH}$ | 0.5 | $W_{TH}^{FCtx}$ | 3.0 | $W_{FCtx}^{TRN}$ | 0.5 |
| $W_{GPi}^{TH}$ | 0.9 | $W_{FCtx}^{STN}$ | 1.0 | $W_{FCtx}^{D1}$ | 0.1 | $W_{FCtx}^{D2}$ | 0.1 | $W_{FCtx}^{FS}$ | 0.001 |
| $I_{D1}$ | $-0.1$ | $I_{D2}$ | $-0.1$ | $I_{STN}$ | 0.3 | $I_{GPi}$ | 0.3 | $I_{GPe}$ | 0.3 |
| $I_{Th}$ | 0.1 | $W_{Input}^{D1/D2}$ | 0.9 | $W_{Input}^{FS}$ | 0.009 | $W_{Input}^{FC}$ | 0.28 | | |

**Supplementary Table 2.** Parameters of the BG model in the color loop.

| | | | | | | | | | |
|---|---|---|---|---|---|---|---|---|---|
| $N$ | 3 | $\tau$ | $10ms$ | $\tau_{STN}$ | $5ms$ | $\tau_{FS}$ | $5ms$ | $\tau_{FC}$ | $10ms$ |
| $\tau_{TH}$ | $5ms$ | $\tau_{TRN}$ | $5ms$ | $\gamma$ | 0.2 | $W_{GPe}^{D2}$ | 1.0 | $W_{D2}^{GPe}$ | 0.4 |
| $W_{GPe}^{D1}$ | 1.0 | $W_{D1}^{GPe}$ | 0.4 | $W_{GPe}^{FS}$ | 0.05 | $W_{FS}^{D1}$ | 0.5 | $W_{FS}^{D2}$ | 0.5 |
| $W_{STN}^{GPe}$ | 0.7 | $W_{GPe}^{STN}$ | 0.45 | $W_{GPe}^{GPi}$ | 0.08 | $W_{STN}^{GPi}$ | 0.7 | $W_{D1}^{GPi}$ | 0.4 |
| $W_{TRN}^{TH}$ | 0.35 | $W_{TH}^{TRN}$ | 0.35 | $W_{FCtx}^{TH}$ | 0.4 | $W_{TH}^{FCtx}$ | 3.0 | $W_{FCtx}^{TRN}$ | 0.35 |
| $W_{GPi}^{TH}$ | 0.7 | $W_{FCtx}^{STN}$ | 0.58 | $W_{FCtx}^{D1}$ | 0.01 | $W_{FCtx}^{D2}$ | 0.01 | $W_{FCtx}^{FS}$ | 0.01 |
| $I_{D1}$ | $-0.1$ | $I_{D2}$ | $-0.1$ | $I_{STN}$ | 0.5 | $I_{GPi}$ | 0.1 | $I_{GPe}$ | 0.1 |
| $I_{Th}$ | 0.1 | $W_{Input}^{D1/D2}$ | 0.99 | $W_{Input}^{FS}$ | 0.09 | $W_{Input}^{FS}$ | 0.28 | | |

**Supplementary Table 3.** Parameters of the STT model.

| | | | | | | | | | |
|---|---|---|---|---|---|---|---|---|---|
| $\tau$ | $5ms$ | $\tau_{Sat}$ | $100ms$ | $\epsilon_{OPN}$ | 0.1 | $\epsilon_{trig}$ | 0.4 | $\epsilon_{stop}$ | 0.5 |
| $W_{SCi}^{LLB}$ | 0.15 | $W_{OPN}^{Mot}$ | 10.0 | $W_{OPN}^{BN}$ | 40 | $W_{Mot}^{Int}$ | 0.05 | $W_{Sat}^{Mot}$ | 6.0 |
| $W_{BN}^{TN}$ | 0.05 | $W_{BN}^{MN}$ | 1.52 | $W_{MN}^{\theta}$ | 4.07 | | | | |

**Supplementary Table 4.** Parameters of the SC integration.

| | | | | | | | | | |
|---|---|---|---|---|---|---|---|---|---|
| $\tau$ | $2ms$ | $W_{SCs}^{SCi}$ | 0.5 | $W_{FEF}^{SCi}$ | 0.3 | $W_{V4\|IT}^{SCi}$ | 0.3 | $W_{SCi^{in}}^{SCi}$ | 0.8 |
| $W_{BG_{amp}}^{SCi}$ | 0.2 | $W_{BG_{inhib}}^{SCi}$ | 3.5 | $W_{SG_{inhib}}$ | 40 | $GPi\|SNR_{rest}$ | 0.25 | $SNR_{rest}$ | 0.25 |

**Supplementary Table 5.** Parameters of the AC modules.

| | | | | | |
|---|---|---|---|---|---|
| $\gamma_{spatial}$ | 0.995 | $\eta_{spatial}$ | 0.00007 | $\lambda_{spatial}$ | 0.95 |
| $\gamma_{color}$ | 0.995 | $\eta_{color}$ | 0.048 | $\lambda_{color}$ | 0.95 |
